# The Appalbees menu: a multiyear, multilocus metagenetic assessment of pollen foraging by Appalachian *Bombus affinis* workers

**DOI:** 10.7717/peerj.20284

**Published:** 2026-01-12

**Authors:** Robert S. Cornman, Mark J. Hepner, Clint R.V. Otto

**Affiliations:** 1 Fort Collins Science Center, U.S. Geological Survey, Fort Collins, CO, United States of America; 2 Metamorphic Ecological Research & Consulting, Alonzaville, VA, United States of America; 3 Northern Prairie Wildlife Research Center, U.S. Geological Survey, Jamestown, ND, United States of America

**Keywords:** Pollen metabarcoding, *Bombus affinis*, Rusty-patched bumblebee, Plant-insect interactions, Pollination, Biomonitoring

## Abstract

**Background:**

Detailed studies of foraging behavior are needed for scientific management of the endangered rusty-patched bumblebee (*Bombus affinis*) in the disjunct and ecologically differentiated habitats it presently occupies. Current knowledge gaps hinder recovery planning but are challenging to redress through direct observation of rare interactions in the field.

**Methods:**

We used genetic metabarcoding to characterize the taxonomic composition of pollen collected by *B. affinis* workers in the Appalachian mountains of Virginia and West Virginia from 2021–2023. We developed a custom sequence database of the regional flora and compared results for two independent genetic loci, internal transcribed spacer 1 and internal transcribed spacer 2 (ITS1 and ITS2).

**Results:**

While ITS2 consistently detected more plant diversity, results from the two loci were broadly concordant with a few notable exceptions. The plant genera *Hydrangea*, *Actaea*, *Rhododendron*, *Tilia*, and (unexpectedly) *Laportea* were prominent in midsummer samples, with *Rubus* a consistent contributor in late spring and early summer. Pea flowers (family Fabaceae) were relatively infrequent but the genera *Securigera* and *Trifolium* were detected before the *Hydrangea* bloom and again in late summer afterwards. The diversity of forage plants was highest in late summer, driven primarily by various genera of Asteraceae. Comparing the current data with previous work indicates regional differentiation in forage plants between Appalachia and the upper Midwest, but also allows ‘consensus’ forage sources that are supported by multiple lines of evidence and shared between regions to be tabulated. These results should help managers focus survey efforts for this endangered species and plan habitat enhancements.

## Introduction

The rusty-patched bumblebee, *Bombus affinis*, is a federally listed species with an historical range spanning from the northern Great Plains eastward to Quebec and southward to the southern Appalachian Mountains. Multiple interacting factors likely contributed to population decline, including pathogens, increased interspecific competition, agrichemicals, and habitat loss (Goulson et al., 2015; Smith et al., 2021). While these stressors continue to persist on the landscape, a potential counterbalance is to improve the nutritional value of *B. affinis* habitat (Vaudo et al., 2015), particularly during critical periods of the life history (Becher et al., 2024). Nutrition is an important component of stress tolerance that has been shown effective in the management of other Apidae (*e.g.*, Huang, 2012; Archer et al., 2014; Azzouz-Olden, Hunt & De Grandi-Hoffman, 2018). Accordingly, multiple studies have sought to better characterize floral resource availability (Mola et al., 2021) and use (Wood et al., 2019; Simanonok et al., 2021; Wolf et al., 2022; Simanonok et al., 2024) by *B. affinis* to inform habitat prioritization and resource seeding. These studies indicate that *B. affinis* is a generalist forager that does not strongly differ from other generalist *Bombus* taxa, but contemporary work has necessarily been restricted in time and space. In particular, the disjunct clusters of extant *B. affinis* populations currently known, in the upper Midwest and in the Appalachian mountains of Virginia and West Virginia, have received unequal attention. Genetic differentiation between these two population clusters is consistent with isolation-by-distance rather than ecotypic or subspecific divisions, indicating they are likely relics of an historically interbreeding population rather than long isolated or ecologically differentiated groups (Mola et al., 2024). Yet the two population clusters may nonetheless differ in resource utilization due to regional differences in flora, local land-use patterns, or habitat availability (Hepner et al., 2024). Thus, distinct regional approaches to habitat management may be required and should be based on direct assessments of local resource utilization.

Methods for assessing resource utilization include direct observation of flowers visited and taxonomic characterization of pollen resources acquired, the latter using either quantitative palynology or genetic metabarcoding. Genetic metabarcoding involves the high-throughput sequencing of taxonomically informative genetic loci to assess the proportions of plant taxa in bulked pollen, whereas palynological microscopy is used to count proportions of pollen morphotypes using specialized keys and reference samples. These methods have various strengths and weaknesses. For example, visual observation can potentially differentiate foraging for nectar (the most common foraging activity (Spaethe & Weidenmüller, 2002)) *versus* pollen, but surveyors cannot quantify the relative contributions of different pollen sources (the main source of lipid and protein in the bee diet). Visual surveys are further constrained by the rarity and potential ambiguity of direct observations and the accessibility of relevant habitat, although these obstacles are being eased by the increasing use of digital photographs to preserve and then classify pollinator interactions after the fact (Wolf et al., 2022; Smith et al., 2024). The two methods that taxonomically bin foraged pollen often show good agreement for the more common plant taxa in a sample, with greater overall diversity generally detected by genetic metabarcoding (Smart et al., 2017; Macgregor et al., 2019; Richardson et al., 2021; Simanonok et al., 2023, but note Carneiro de Melo Moura et al., 2022 for a counterexample). Microscopy often has reduced taxonomic resolution (Smart et al., 2017), whereas proportions inferred by genetic metabarcoding can be skewed by various molecular biases (Gold et al., 2023). While genetic metabarcoding is increasingly used due to the potential for high throughput at low relative costs (Macgregor et al., 2019), validation with external data and across genetic loci remain important for strengthening conclusions and reducing management uncertainty.

This study used genetic metabarcoding to characterize pollen foraging behavior of *B. affinis*, focusing on Appalachian management areas for which contemporary pollen data were previously lacking. Corbicular pollen samples were collected from workers actively foraging on specific host plants. Potential host plants were initially targeted based on accessibility and general attractiveness to *Bombus*; when *B. affinis* was found visiting a particular taxon, search effort was focused on that taxon while in bloom. Host plants were therefore not randomly sampled but were instead skewed in response to field conditions and prior experience (further detailed in Hepner et al., 2024). To increase confidence in metabarcode inferences, a custom database of the regional flora was generated and vetted. Metabarcode data were then generated for two primer sets targeting the internal transcribed spacers (ITS) of the ribosomal RNA locus (a multicopy nuclear gene). The objectives of the study were to: (1) assess whether a host-targeted survey approach yielded adequate non-host plant diversity, (2) determine whether inferred pollen compositions were comparable between the two genetic loci, and (3) evaluate whether either locus had a performance advantage in terms of taxonomic resolution, diversity detected, and host species detected. Additionally, we compare the forage plants identified here with those identified in other studies to generate a consensus list of taxa that support *B. affinis* populations across its range.

## Materials & Methods

### Sampling

Appalachian pollen samples (*n* = 86) were collected by Metamorphic Ecological Research and Consulting, LLC, spanning June–August of 2021–2023. Sampling in 2021 and 2022 began in late June whereas in 2023 a small number of samples were obtained in early June. Three additional samples from Illinois and Wisconsin were also included to buttress comparisons between Midwestern and central Appalachian forage diversity; these samples were collected in August 2023 by Environmental Solutions & Innovations, Inc. Collections were made under the following permits where required by state and federal agencies: eS61005D (US Fish and Wildlife Service [USFWS]); 2021.085, 2022.113, 2023.196 (West Virginia Department of Natural Resources); 4200 (Monongahela National Forest); 2600, 2670 (George Washington and Jefferson National Forests); ES02373A-16 (USFWS).

Appalachian survey locations were chosen with the goals of documenting populations not previously known as well as for feasibility of bee identification and capture; these considerations favored roadsides, fields, and forest edges and are further detailed in Hepner et al. (2024). *B. affinis* workers were netted while foraging on a host plant identified at least to genus, with the exception of a single individual opportunistically netted in flight. Bees were chilled on ice, allowing a single corbicular pollen ball to be removed with forceps and placed in a labeled microcentrifuge tube prior to releasing the bee. Forceps were cleaned with alcohol between sampling events. Sampled bees were photographed for verification and vouchering. Appalachian samples were stored dry whereas Midwestern samples were stored in ethanol (storage method has minimal effect on taxonomic characterization (Quaresma et al., 2021)).

The Midwestern samples were strictly opportunistic samples that were provided to us by the USFWS and had been collected during permitted *B. affinis* surveys unrelated to the Appalachian survey described here. While the three samples do not represent any coherent effort to obtain pollen samples from a specific region or environment, they nonetheless contain information about *B. affinis* forage behavior. Any comparison beyond descriptive statistics between the two regions is obviously constrained by the large difference in sample size; here we limit our comparison to three topics: (1) the potential effects of sample-level stochasticity on concordance between internal transcribed spacer 1 (ITS1) and internal transcribed spacer 2 (ITS2) detections (*i.e.,* concordance in a small *versus* a large sample set) (2) calculating the *proportional* overlap that the current Midwestern and Appalachian pollen samples have with external data sets, particularly the large Midwestern survey of Wolf et al. (2022), and (3) including the Midwestern samples and the Appalachian samples as two of seven independent data sources, including five previously published data sets, from which a ‘consensus’ forage list was generated. This list was based only on positive detection of a genus in at least one data set from each region (discussed further below).

### Library preparation and sequencing

Pollen DNA was extracted using the Plant DNeasy kit (Qiagen) with mechanical disruption in lysis buffer. Disruption was performed with MP Biomedicals lysing matrix A in two mL centrifuge tubes, shaken with a FastPrep-24 5G Bead Beating Grinder (MP Biomedicals) for 40 s at 6 m/s. Two primer sets were used to amplify the ITS1 and ITS2 genetic loci *via* polymerase chain reaction (PCR). The ITS1 primers consisted of primer ITS5A from Stanford, Harden & Parks (2000) and the reverse complement of primer ITS-3p62plF1 from Kolter & Gemeinholzer (2021). The ITS2 primers consisted of ITS-3p62plF1 and ITS-4unR1 from Kolter & Gemeinholzer (2021). These locus designations are approximate, however, as the ITS-3p62plF1 primer is located near the 3′ terminus of the ITS1 sequence. Thus, the “ITS1” amplicon lacks a short terminal region of ITS1 sequence that is included in the “ITS2” amplicon (along with the highly conserved 5.8S rRNA sequence).

The base amplicon was generated from genomic DNA for each locus using a low cycle-number “preamplification” reaction with the primers cited above. PCRs consisted of 3 µl DNA solution, 0.2 µl Promega GoTaq, five µl Promega 5X reaction buffer, 2.5 µl primer mixture (one µM stock concentration), 1.5 µl Promega magnesium chloride solution, 2.5 µl deoxyribonucleotide triphosphates (0.25 mM stock concentration) and 10.3 µl molecular-grade water. Preamplification PCRs were performed in triplicate and pooled to minimize stochastic variation, then cleaned of residual reagents using the Qiagen Qiaquick 96-well PCR Purification kits for vacuum or centrifuge. Reactions were run in 96-well thermocyclers with heated lids, using the following protocol: an initial denaturing step at 95 °C for 2 min, followed by 12 cycles of 95 °C for 30 s, 50 °C for 30 s, and 72 °C for 30 s. A final extension step of 72 °C was implemented for 2 min, followed by an indefinite hold at 4 °C.

Cleaned preamplification products were then input into a second PCR to append exogenous adapter sequences for sequencing on the Illumina platform, using primers modified as described in the manufacturer’s protocols (Illumina document 15044223 Rev. B, available at https://support.illumina.com/documents/documentation/chemistry_documentation/16s/16s-metagenomic-library-prep-guide-15044223-b.pdf). Four equimolar variants of the modified primers were used in this reaction, the minimal primer described in the Illumina protocol and three additional primers containing one to three completely degenerate bases inserted between the Illumina adapter and the locus-specific sequence. These additional linker bases generate phase diversity (Wu et al., 2015) that improves the calibration of the sequencing run by moderating signal intensity at highly conserved positions within a barcode. Reactions consisted of three µl cleaned preamplification product, 2.5 µl of 10 µM primer mixtures, 12.5 µl KAPA2G DNA polymerase reaction mix (Roche), 0.25 µl bovine serum albumin solution, and 6.75 µl molecular grade water. Thermocycler conditions were as described above except the cycle number was increased to 24 and the extension time was increased to 40 s. These reactions were performed in duplicate, then pooled and cleaned as described above.

The final amplification step of the amplicon libraries appended sample-specific indexing oligonucleotides using Illumina Nextera CD combinatorial dual index kits. These reactions followed the manufacturer’s protocol without modification and were cleaned of residual reagents as described above. Prior to the indexing PCR, amplicons of the two primer sets were quantified with a Qubit Model 2 fluorometer and Broad-Range DNA Assay (Invitrogen) and then pooled to approximate equal concentrations within samples. Libraries were then diluted as per manufacturer’s protocol and sequenced with Illumina MiSeq v.3 cartridges to generate 250-bp paired-end reads. Sequencing output for biological samples was deposited in the Sequence Read Archive (SRA) of the National Center for Biotechnology Information (NCBI) under BioProject PRJNA1235776.

### Reference database generation

A regional reference database was generated to support the assignment of metabarcode sequences to plant taxa of origin. The database was generated by subsetting the PLANiTS ITS sequence database (Banchi et al., 2020) to include only genera listed to occur in the sampling region in the USDA Plants database (available at https://plants.usda.gov/). The sampling region was broadly construed so that the database would be generalizable to similar applications in the Mid-Atlantic and Appalachian regions of the US. Furthermore, because plant distributions are imperfectly known at the state level, overly narrow geographic limits can exclude taxa that are actually present. Therefore, we selected all genera listed as present within any of the following ten US states: Virginia, West Virginia, Maryland, Pennsylvania, North Carolina, Tennessee, Ohio, Kentucky, Delaware, and New Jersey. PLANiTS sequences belonging to these genera were selected regardless of specific epithet, provided the species was recognized in the NCBI taxonomic database (as official taxon or recognized synonym). Geographically unexpected species within geographically expected genera can therefore be included in the database; this is desirable because invasive and horticultural congeners are often absent from geographic checklists and because including many representatives of a genus should improve discrimination at the genus level, the primary level of analysis in this study (discussed further below).

Database sequences were screened for fungal contamination, which can occur with these primers (*e.g.*, Cornman et al., 2015), by classifying them with the UNITE fungal database (Kõljalg et al., 2019), which is pre-formatted for SINTAX (Edgar, 2016) and was accessed on 02/19/25. This contamination screen resulted in the removal of two reference sequences. An additional 31 reference sequences were obtained from NCBI representing taxa that were indicated by the data but absent from the PLANiTS database (*i.e.,* by searching abundant unassigned operational taxonomic units (OTUs) against the comprehensive nucleotide database of NCBI). Two taxonomic names were replaced with accepted synonyms to avoid artificial family-level assignments: *Menziesia pilosa* was equated to *Rhododendron pilosum* (Craven, 2011) and *Chrysoma pauciflosculosa* equated with *Solidago pauciflosculosa* (Semple et al., 2023). Reference sequences shorter than 250 bp were then removed as insufficiently complete, and identical sequences with the same taxonomy were de-replicated with the “derep_fulllength” command of vsearch *v.* 2.21.0 (Rognes et al., 2016). The final reference database included 6,107 sequences representing 1,125 genera and 2,751 species (File S1); sequences were converted to uppercase if necessary because lowercase characters can be interpreted as masked by some software. ITSx *v.* 1.1.3 (Bengtsson-Palme et al., 2013) and the bbmap package (available at https://sourceforge.net/projects/bbmap) were used to extract ITS sequences and expected amplicon sequences, respectively, from the reference database for inspection, such as to determine whether taxa detected at only one primer set were fully represented in the database.

### Bioinformatic processing

Read pairs were trimmed of low-quality bases using the bbduk program of the bbmap package, using a quality trim value of 10 (Phred-scaled). Trimmed read pairs were merged with vsearch specifying a minimum overlap of 65 bp and a maximum percent difference of 8% within the overlap. Unmerged pairs and merged pairs less than 300 bp in length were discarded (the mode amplicon lengths were 329 bp and 345 bp for ITS1 and ITS2 primer sets, respectively). OTUs were generated by clustering merged reads at 99% with vsearch using similarity definition “1” of that program. Primer sequences were removed from the OTUs prior to taxonomic assignment with bbduk using a search kmer value of 11.

A taxonomy was assigned to each OTU with the vsearch SINTAX command using the reference database described above. The SINTAX method assigns a confidence score to each rank of the assignment; we assigned the OTU the lowest rank of the SINTAX assignment hierarchy that had a confidence score of at least 0.9 (possible range is 0–1 with higher scores indicating higher confidence). The number of reads attributed to each taxon within each sample was the sum of cluster sizes for all OTUs with the same assignment (multiple OTUs may have the same taxonomic assignment within a single sample). Unfiltered taxon counts and counts filtered, scaled to counts per million (cpm), and aggregated at the genus level are provided in File S2. By policy, the raw data are also contained within a U.S. Geological Survey (USGS) data release (Cornman & Otto, 2025). As the analyses presented here are based on prevalence and Shannon’s diversity, which depend only on proportions, no log-ratio transformation was performed to open the inherently closed, compositional nature of the data (reviewed by Gloor et al., 2017).

A common filtering practice (Alberdi et al., 2018; Drake et al., 2022) is to censor low count values within each sample (*i.e.,* convert those cells of the taxon matrix to missing or zero) rather than count them as detections, because low taxon counts within individual samples may arise through erroneous index sequences (referred to as ‘demultiplex error’ or ‘cross-talk’, *e.g.*, Edgar, 2018; MacConaill et al., 2018). For both loci, sequence counts in the negative control sample were consistent with known rates of demultiplex error for Nextera eight-base dual indexes (MacConaill et al., 2018) for both loci, indicating no systemic contamination occurred (Fig. S1). An additional consideration is that taxa that comprise a small proportion of a sample total could potentially arise from ‘natural’ contamination, *i.e.,* wind- or insect-mediated transfer of heterospecific pollen. The frequency and mechanisms of heterospecific pollen incorporation in *B. affinis* corbicular pollen loads are unknown and likely contingent on factors such as weather, plant community, and insect community (discussed further below). Given our primary goal of identifying common pollen forage sources relevant to the management of *B. affinis* habitat and the ambiguity as to whether plants at low abundance in a sample were actively foraged, we limited positive detections in a sample to those plant taxa with at least ten sequence reads and constituting at least 0.1% of the total plant-assigned sequence reads. Counts in samples for taxa with nonzero counts in the negative control were not further adjusted or removed, as the rationale for these thresholds includes mitigating the cross-talk detected in the negative control.

After this censoring, counts were aggregated at the genus level for further analyses. Because of the high number of rare genera detected, some analyses were limited to genera detected in at least three samples (see ‘Results’). Shannon’s diversity index was estimated with PAST *v.* 4.17 (Hammer, Harper & Ryan, 2001), which was also used to perform nonparametric analyses of variance.

Overlap with genera detected in previous studies of *B. affinis* foraging was assessed by referencing Table S2 of Simanonok et al. (2021), Table 1 of Wolf et al. (2022), Table 2 and Table 3 of Simanonok et al. (2024), and Figure 2 of Hepner et al. (2024), as summarized in File S3. Taxa above genus rank in those references were excluded. Wood et al. (2019) examined corbicular pollen loads of multiple *Bombus* species including *B. affinis*, but did not list these by species and thus could not be included in this comparison.

### Evaluation of bioinformatic parameters with simulated data

To evaluate merge parameters and taxonomic assignment accuracy, paired 250-bp sequence reads were simulated for the ITS2 locus with ART (Huang et al., 2012) to generate 1000X coverage of a modified version of the reference database. References were oriented uniformly and trimmed so that the ITS2 forward primer sequence was 5′; the 3′ end was not specifically trimmed to the reverse primer so that partial reference sequences could be included. References with greater than 1% ambiguous characters or lacking an explicit species-level taxonomic assignment were excluded. Illumina MiSeq amplicon error profiles were modeled by selecting settings “MSv3” and “amp” in ART. Simulated reads were then trimmed, merged, and assigned as described above but with key parameters systematically adjusted.

Merge rates for simulated reads were determined for 54 combinations of overlap and maximum percent mismatch values, in which the overlap incremented from 60 to 100 in steps of 5 and the mismatch value incremented from 5 to 10 in steps of 1. Little difference was seen among these combinations, with merge rates greater than 98% for all parameter combinations.

Taxonomic error rates were calculated for three SINTAX scores: 0.8, 0.85, and 0.9 (Fig. S2). At a threshold of 0.9, 92.5% of species assignments were correct and 0.26% incorrect, with the balance unassigned. At a threshold of 0.8, 95.2% of species assignments were correct and 0.37% incorrect, with the balance unassigned. At the genus level, greater than 99% of assignments were correct at all thresholds and error rates were an order of magnitude lower than at the species level (ranging from 0.0259%–0.0538%). Furthermore, genus-level error rates were concentrated in only a few species. For example, at a threshold of 0.9, the uncommon crop species *Momordia charantia* and two *Centaurea* species accounted for over 90% of all genus-level errors. On the other hand, the simulation results likely underestimate the true error rate because biological samples often contain valid but unrepresented sequence variation for a taxon, and often include taxa that are missing from or misassigned in the reference database. Nonetheless, the simulated data support the bioinformatic parameters chosen and indicate a high-level of confidence in taxonomic assignments when high-quality reference sequences for a taxon are available, at least for the ITS2 locus. The observation that simulated sequences were much more likely to be unassigned than incorrectly assigned suggests that ITS1 sequences assigned at a conservative (high) threshold are also unlikely to generate significant genus-level false positives.

## Results

### Adaptive targeting of focal plants recovers high pollen diversity

**Figure 1:**
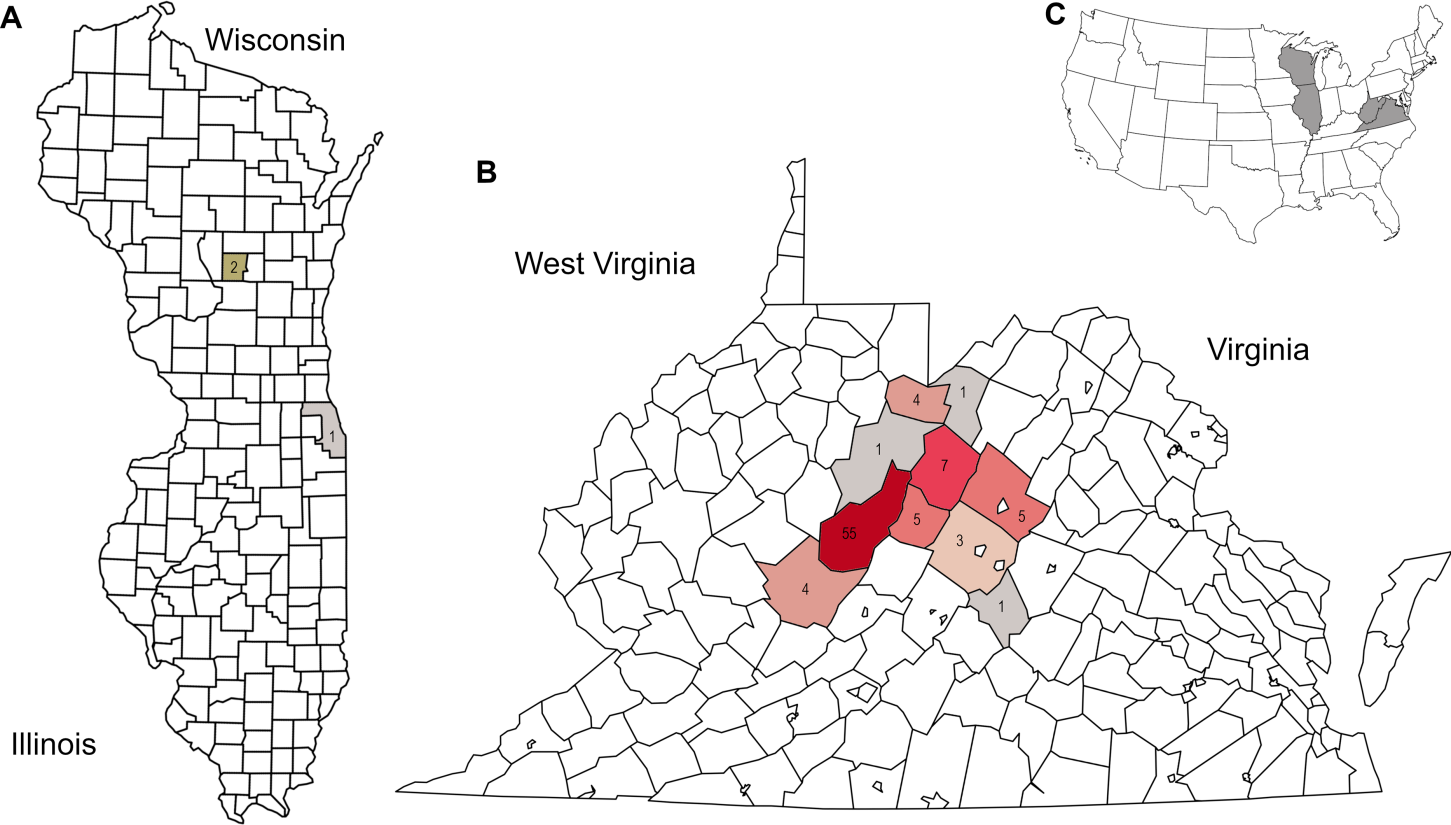
Distribution of corbicular pollen samples by US county of collection, color-coded by number of samples. Precise locations of protected species habitats are withheld. A. Locations of Midwestern samples. B. Locations of Appalachian samples. C. Sampled states shown in context of the contiguous United State.

**Figure 2:**
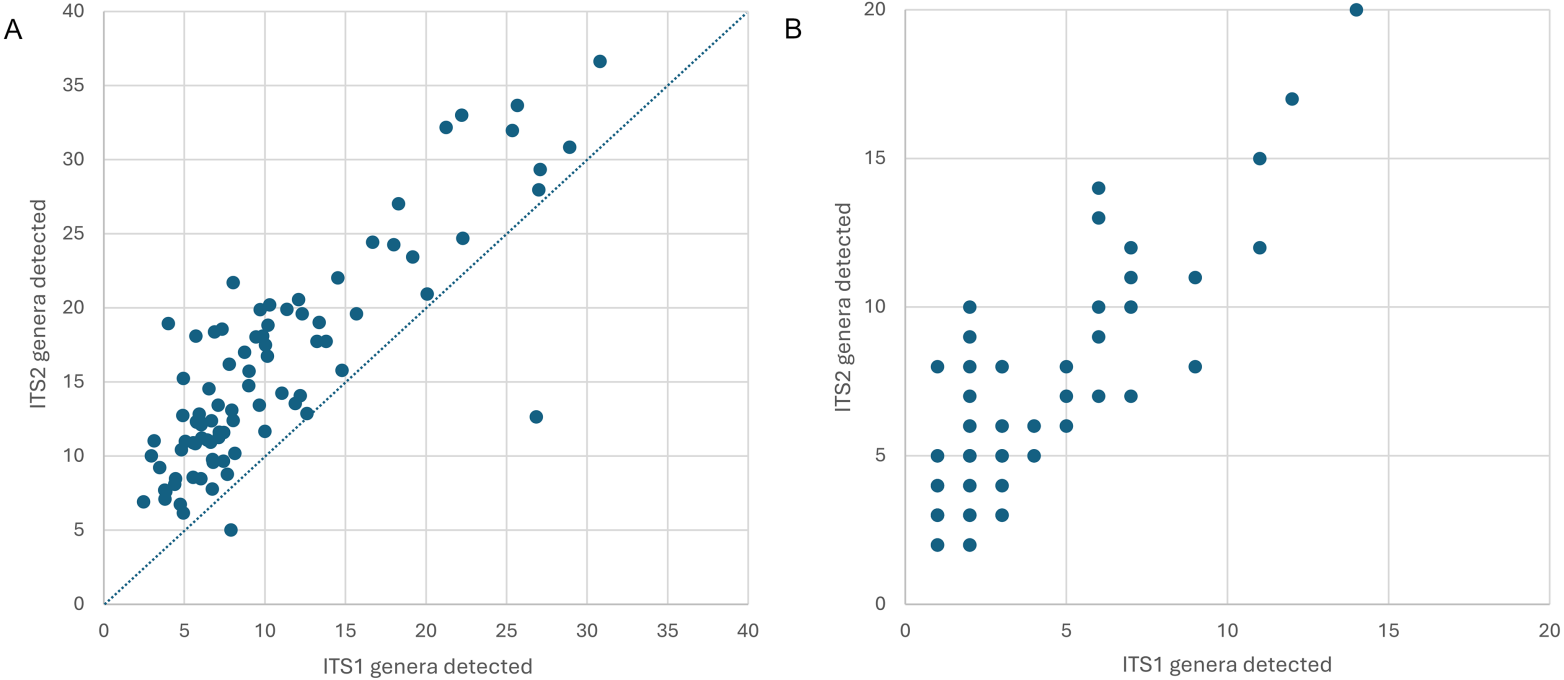
Genus-level plant diversity detected in corbicular pollen samples with two genetic barcode loci. (A) The number of genera expected in 10,000 sequence reads per sample prior to filtering, plotted by locus. The dashed line corresponds to equal richness. These values represent estimated potential diversity given equal efforts. (B) Total genera detected per sample, after filtering. These values represent realized diversity for selected filtering criteria and actual sampling effort (which is inherently unequal among samples). ITS1 = internal transcribed spacer 1 and ITS2 = internal transcribed spacer 2.

Appalachian samples were collected from early June to late August over three consecutive years (2021–2023), necessarily clustered around periods of greater survey effort and greater worker abundance. A total of 89 corbicular pollen samples were sequenced, 86 from Appalachian sites and three from comparison sites in the upper Midwest (Fig. 1). The number of sequences assigned to plant taxa ranged from 87–144,221 (mean 44,385) for ITS1 and 34–84,144 (mean 40,321) for ITS2. Although most sequences were assigned at the species level (84.5% for ITS1 and 76.8% for ITS2), taxonomic assignments are analyzed herein at the genus level to maximize both the amount and robustness of data included (see simulated error rates in Materials and Methods). The minimum number of assigned sequences for inclusion of a sample in analyses was 5,000; this threshold excluded two samples at the ITS1 locus and one sample at the ITS2 locus. After these exclusions, the mean genus-level richness detected per sample was 3.40 (2.72 SD) at ITS1 and 6.08 (3.50 SD) at ITS2. While many of the additional ITS2 detections are taxa with low relative abundance and low prevalence, rarefaction to a fixed sampling effort of 10,000 sequence reads further demonstrates the consistently greater genus diversity detected at the ITS2 locus (Fig. 2).

**Figure 3:**
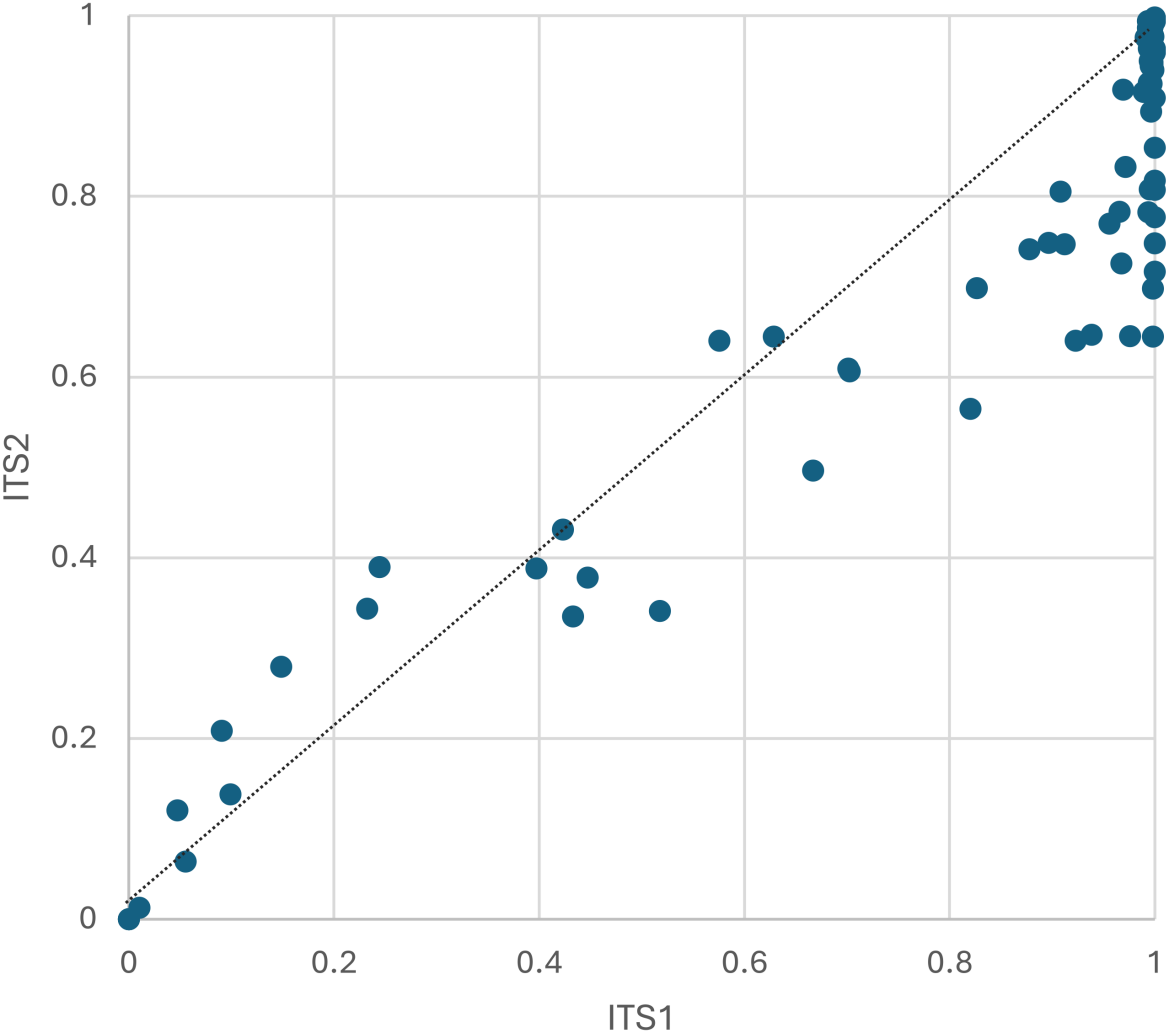
The proportion of plant host sequence reads detected per corbicular pollen sample, by locus. The dashed line serves as a visual reference for equal proportions to highlight differences between loci. ITS1 = internal transcribed spacer 1 and ITS2 = internal transcribed spacer 2.

The proportion of sequences consistent with pollen of the host plant from which the worker was captured was highly variable among samples but overall comprised roughly three-quarters of assigned sequence reads (Fig. 3, File S2). The mean proportion of non-host reads was 0.190 for ITS1 and 0.273 for ITS2. Notably, all four samples taken from bees visiting *Monarda fistulosa* (wild bergamot) failed to yield sequences of that host plant above the threshold detection level for both loci. This is likely a biological rather than methodological phenomenon, as bumblebees are frequently observed robbing *Monarda* of nectar in a manner that precludes contact with anthers (Wolf et al., 2022) and *Monarda* metabarcode sequences have been detected previously from honeybee-collected pollen with comparable primers (Smart et al., 2017; Otto et al., 2020).

### Comparison of plant diversity detected at each locus

**Figure 4:**
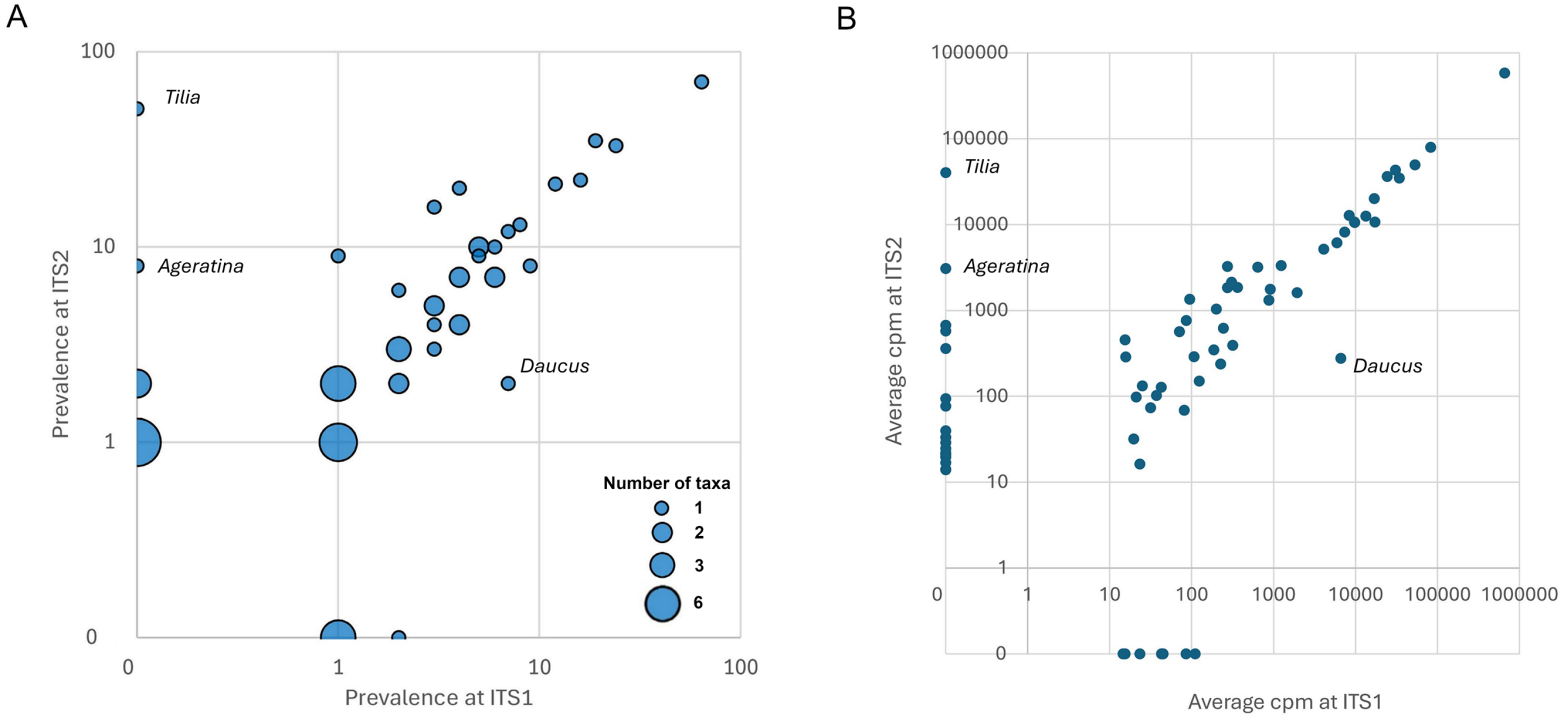
Degree of concordance between ITS1 and ITS2 loci. Discordant genera that are discussed in the text are identified on the figure. All genera had at least one reference sequence spanning the full length of the amplicon except *Ageratina* (marked with magenta), which lacked reference sequence for the ITS1 amplicon. (A) Bubble plot showing prevalence of genera at each locus. The size of points is scaled to the number of genera with a given set of values. (B) Relative abundance of sequence reads for each genus in the raw data set, by locus. For rare taxa, relative abundance is correlated contingent on detection in both data sets, suggesting that detection at only one locus is primarily stochastic rather than ascertainment bias. ITS1 = internal transcribed spacer 1 and ITS2 = internal transcribed spacer 2.

In total, 69 plant genera were detected in Appalachian samples, 45 (65.2%) of which were detected at both loci (Fig. 4A). Seven genera were detected only at ITS1 and 17 genera were detected only at ITS2 (File S2). For the three Midwestern samples, eight of 21 total genera were detected at both loci (38.1%), two were detected only at ITS1 and 11 were detected only at ITS2. The total number of detections summed across samples was 268 at ITS1 and 469 at ITS2.

Many of the differences between loci appear to be due to stochasticity in the detection of rarer taxa *per se* rather than intrinsic biases between loci. For example, five of the genera detected at only one locus in Midwestern samples (*Hypericum*, *Rudbeckia*, *Senecio*, *Impatiens*, and *Ageratina*; Fig. S2) were also detected in Appalachian samples, but in that larger data set three of the five were detected at both loci (*Hypericum*, *Rudbeckia*, and *Impatiens*). Moreover, the single *Ageratina* reference sequence did not include the region amplified by the ITS1 primer set and therefore that genus would likely have been assigned at a higher rank if amplified from samples. More generally, genera detected at only one locus in Appalachian samples mostly had low prevalence at that locus (Fig. 4A), with the exception of *Tilia* (*Ageratina* is uninformative in this regard as it lacked ITS1 reference sequence). While detected at both loci, *Daucus* was notably more prevalent at ITS1 than ITS2.

The two loci were also concordant with respect to relative abundances of sequences attributed to each genus, again declining somewhat for rarer taxa as expected with sampling stochasticity (Fig. 4B). The taxa that strongly differed in prevalence also strongly differed in relative abundance: ITS2 primers amplified much less *Daucus* than ITS1 overall, whereas *Tilia* was abundant in ITS2 sequences but absent in ITS1 sequences. The cpm values for genera were highly correlated when both were detected (Pearson’s *r* = 0.999, *P* = 4.77E−61). While the primer sets were broadly consistent in this regard (with the stated exceptions), consistency does not imply that taxon relative abundances are unbiased, but does suggest that metrics based on relative abundance, such as frequency-based diversity indices, would also be reasonably consistent between the two loci. For example, Shannon’s H was well correlated between the two loci (Pearson’s *r* = 0.753, *P* = 2.8E−7) after removing very low diversity samples (*H* < 0.1 at either locus).

### Forage patterns in Appalachian samples

**Figure 5:**
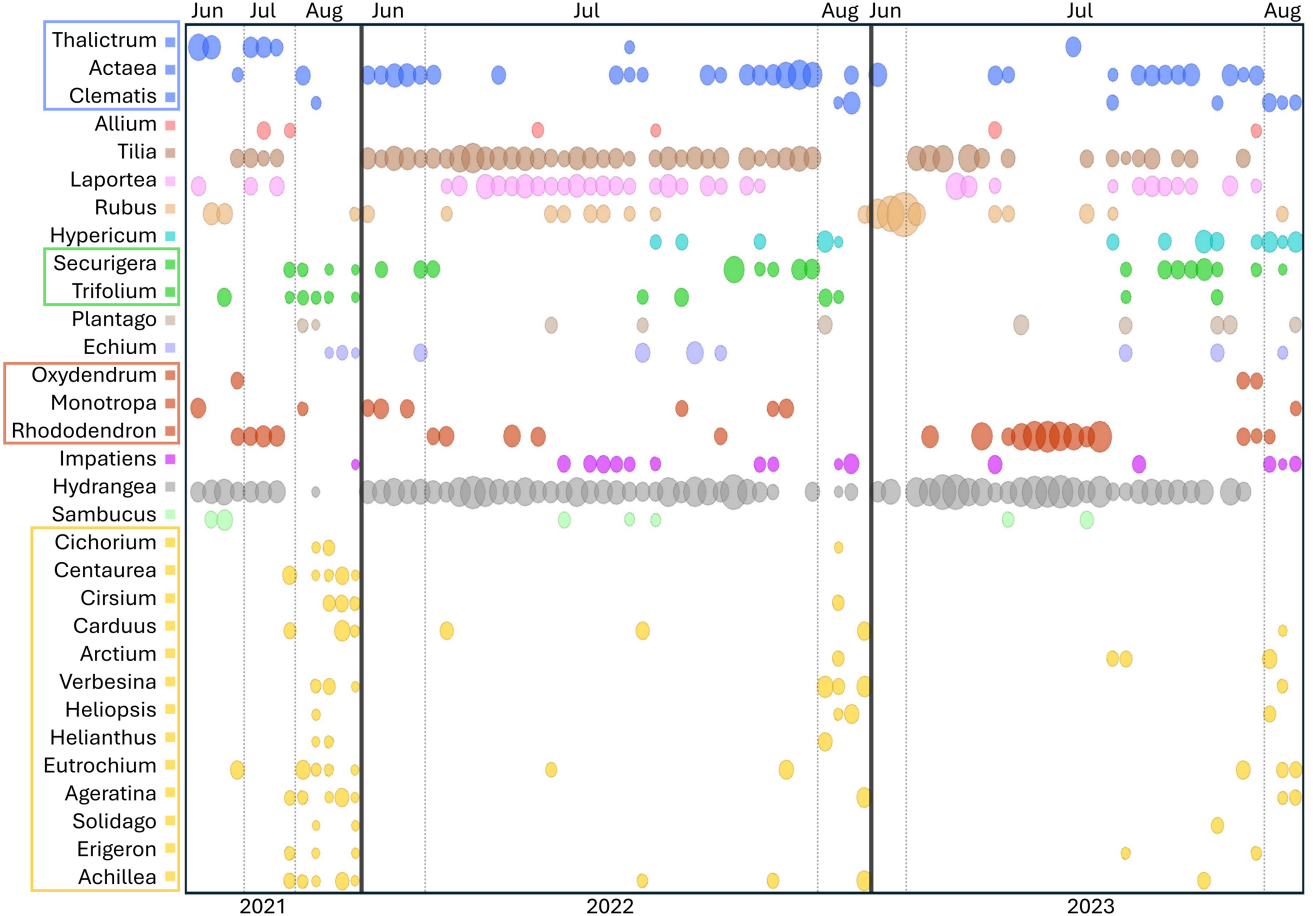
Temporal pattern of genera detected at the ITS2 locus in at least three Appalachian pollen samples. To increase the visibility of low-abundance taxa within a sample, circle areas were scaled to the natural log of relative read abundance before conversion to proportions of the sample total. Light vertical bars separate samples by month, dark vertical bars separate samples by year. Genera within the same family are boxed and share a common color: blue = Ranunculaceae, green = Fabaceae, red = Ericaceae, yellow = Asteraceae. ITS2 = internal transcribed spacer 2.

**Figure 6:**
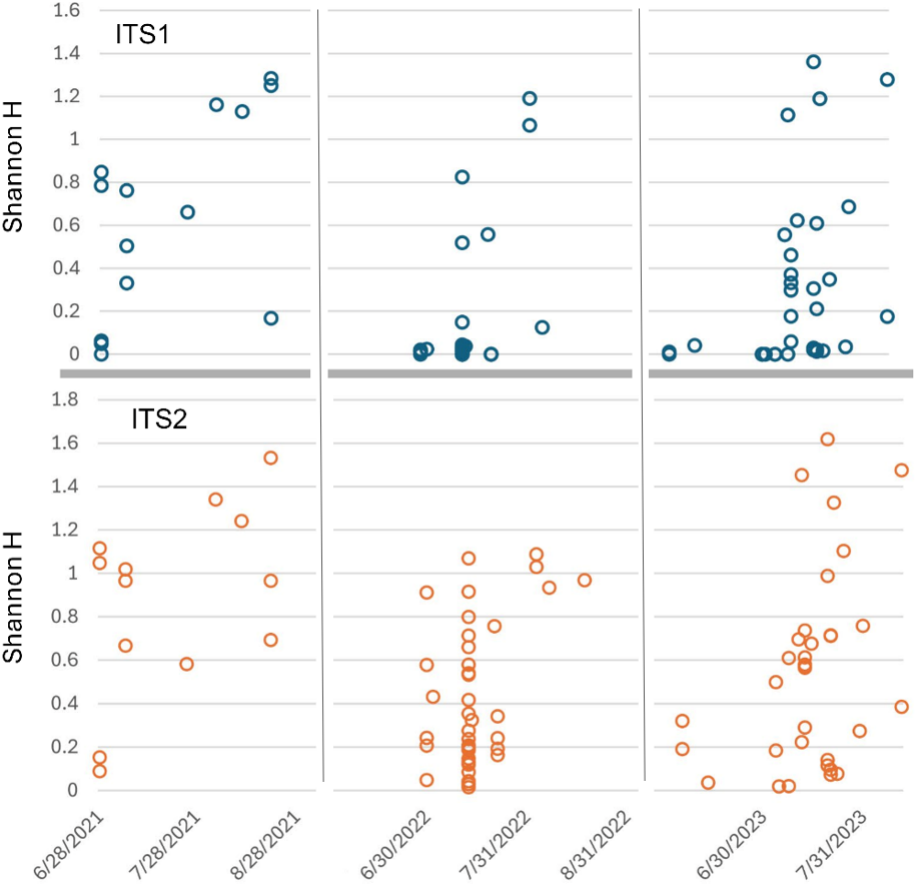
Shannon diversity index (H) for Appalachian samples plotted on a temporal scale and separately by locus. Both loci show generally higher diversity in later samples relative to early ones, particularly August samples. ITS1 = internal transcribed spacer 1 and ITS2 = internal transcribed spacer 2.

Sorting Appalachian samples chronologically (Figs. 5–6) highlighted a forage pattern in which diversity was lower and more consistent in early summer, albeit somewhat confounded by the tendency for more captures during this period, particularly on *Hydrangea* host plants. Note the size of the ellipses in Fig. 5 are natural-log scaled to increase the visibility of proportionally rare taxa, and only taxa detected in at least three samples are shown. Genus diversity was higher and more variable among samples in later summer (Figs. 5–6), a pattern observed each year. When samples were binned by calendar month across years (Fig. S3), median Shannon’s H differed significantly by month (Kruskal–Wallis test, *P* = 0.00031 for ITS1 and *P* = 0.0011 for ITS2). For both loci, August had significantly higher median Shannon’s H than June and July whereas the latter two months did not significantly differ in pairwise comparisons (see Fig. S3 for Bonferroni-corrected *P*-values).

Figure 5 also illustrates a relative paucity of Fabaceae in this data set compared with some other studies (Simanonok et al., 2021; Simanonok et al., 2023; Simanonok et al., 2024) but which is consistent with the lower visitation of Fabaceae by *B. affinis* relative to congeners reported by Wood et al. (2019). Wolf et al. (2022) also noted the relative rarity of observed *B. affinis* visits to several common Fabaceae widespread in Wisconsin, such as *Lotus corniculatus*, *Vicia purpurea*, *Melilotus sp.*, and *Trifolium pratense*. The pea-flower genera *Trifolium* and *Securigera* were nonetheless repeatedly detected in Appalachian samples early in the season and in August after the more dominant forage plants were no longer flowering (Fig. 5). Other late-summer forbs identified as forage sources were predominantly within the Asteraceae family, *e.g.*, *Ageratina*, *Carduus*, *Helianthus*, *Verbesina*, and *Solidago* (Fig. 5).

Shrubs and trees commonly detected in Appalachian pollen forage included *Rubus*, *Rhododendron,* and *Sambucus* as well as an apparent abundance of *Tilia*, with the caveat that the latter was detected only with the ITS2 primers as noted above. *Tilia* is known to be attractive to *Bombus* (Anderson, 1976; Jacquemart et al., 2018) and flowers abundantly in June and July but not in August (Anderson, 1976, https://www.inaturalist.org/taxa/54854-Tilia-americana), consistent with our data (Fig. 5). Interestingly, a potential toxicity of *Tilia* nectar to bees has been reported, particularly in Europe, although effects on bee mortality have been questioned (Koch & Stevenson, 2017; Jacquemart et al., 2018; Lande et al., 2019). While the majority of *Tilia* sequence reads were assigned at the genus level (File S2), those assigned at the species level were almost exclusively *T. americana* (basswood), which has not been reported to have a negative effect on *Bombus* pollinators to our knowledge. *Sorbus*, *Oxydendrum*, and *Kalmia* were only infrequently detected (File S2), however the overlap between survey efforts and flowering was likely low for the latter two taxa based on phenology records in the Maryland Biodiversity Project online database (https://www.marylandbiodiversity.com/). Another common genus of shrubs in the region, *Vaccinium*, potentially overlapped in flowering with early June survey dates but went undetected in our data. *Vaccinium* is known to be important forage for many bumblebee species (Cane et al., 1985; Jacquemart, 1993; Lichtenberg & Graves, 2023), although Jacquemart (1993) found that most bumblebee visits in similar European habitat (the Ardennes) were by queens.

### Comparisons with other *B. affinis* forage surveys

While the present study does not systematically review historical *B. affinis* forage plants documented in the literature, the genera identified here (combined across loci) can be compared to several recent surveys of contemporary foraging patterns that employed various observational and pollen-binning methods. These additional studies include two metabarcoding data sets (Simanonok et al., 2021; Simanonok et al., 2024), the palynological component of Simanonok et al. (2024), the observation- and imagery-based compilation of Wolf et al. (2022), and the observational survey of Hepner et al. (2024). Hepner et al. (2024) and the present study represent Appalachian surveys, whereas Midwestern surveys include Wolf et al. (2022) and Simanonok et al. (2024), and the present Midwestern samples. Simanonok et al. (2021) included museum samples from across the northern tier of the historical range and overlaps spatially with the Midwestern data sets but not with the Appalachian data sets.

Overall, 180 plant genera were detected at least once in these studies, but only 77 of these were found in more than one data set (File S3). As with the between-locus comparison, many of the genera unique to individual studies are likely a consequence of rarity *per se*. For example, of the 28 genera unique to the current Appalachian data set, 23 (82.1%) were detected in three or fewer samples. Furthermore, the number of unique genera detected appeared to scale with the total number of genera detected overall rather than relate to methodology (Fig. S4). Other sampling and biological factors likely to contribute to differences among studies include geography, season, sampled habitats, scale, and ascertainment biases (*e.g.*, visual methods may be biased against trees, as noted by Wolf et al., 2022). Regional differentiation is indicated by the fact that fewer than half of the genera detected in Appalachian samples (29 of 69, or 42.0%) were also listed as Midwestern forage plants by Wolf et al. (2022) from surveys and digital imagery (File S3). In contrast, 15 of 21 genera (71.4%) detected from the three Midwestern samples (with identical methodology) were also identified by Wolf et al. (2022).

While it is difficult to interpret differences in genera detected among studies for the above reasons, it is possible to identify a ‘consensus’ list of forbs identified at least once by both pollen taxonomy and visual observation, and at least once in both Midwestern and Appalachian data sets. These taxa (Table 1) can be viewed as high-confidence forage plants visited by worker *B. affinis* that could be targeted with seed mixes or other interventions. The frequency of use of those floral resources should then be readily quantifiable by multiple independent means in subsequent monitoring. Of the 36 listed genera, 32 (88.9%) were found in the current Appalachian data set and 33 (91.7%) were found visually by Wolf et al. (2022), indicating the two approaches were comparably effective at monitoring this core set of commonly visited forbs (File S3). The remaining data sets individually detected 30.5–63.8% (mean 50.5%) of the genera in Table 1. Note *Rhododendron* and *Hydrangea* are only minimally present in the upper Midwest based on USDA Plants data (https://plants.usda.gov/plant-profile/RHODO, https://plants.usda.gov/plant-profile/HYDRA), but both genera include common horticultural plants likely present in that region and are also widespread throughout the remaining historical range. Importantly, the systematics of some listed genera have been subject to revision, such that genetic and distributional records may not fully align with current treatments, for example genus *Rhododendron* (Craven, 2011) and tribe Eupatorieae (Robinson & King, 1985), which includes *Eutrochium*, *Eupatorium*, and *Ageratina*.

**Table 1:** Plant genera consistently detected across management regions and survey methods. Listed genera were identified by both pollen taxonomy and visual observation, and from both upper Midwestern and Appalachian samples reported since 2022. Genera also identified in a multi-region metabarcoding survey of historical (museum) samples are marked with an asterisk (see text for details). ITS2 = internal transcribed spacer 2.

Genus	Family	Representative common names	Prevalence in Appalachian samples (ITS1)	Prevalence in Appalachian samples (ITS2)
Actaea*	Ranunculaceae	bugbane, cohosh	0.286	0.388
Ageratina*	Asteraceae	snakeroot	0^†^	0.094
Allium*	Amaryllidaceae	wild onion	0.024	0.071
Anemone*	Ranunculaceae	anemone	0	0.012
Arctium	Asteraceae	burdock	0.048	0.047
Campanula	Campanulaceae	harebell	0.012	0.024
Carduus	Asteraceae	thistle	0.071	0.082
Centaurea*	Asteraceae	knapweed	0.036	0.059
Cichorium*	Asteraceae	chicory	0.024	0.035
Cirsium*	Asteraceae	thistle	0.048	0.047
Daucus	Apiaceae	wild carrot	0.083	0.024
Eryngium*	Apiaceae	eryngo	0	0
Eupatorium*	Asteraceae	snakeroot, Joe-Pye weed	0.012	0.024
Euthamia	Asteraceae	goldentop	0.012	0.000
Eutrochium*	Asteraceae	Joe-Pye weed, throughwort	0.071	0.118
Helianthus*	Asteraceae	sunflower	0.036	0.035
Hydrangea*	Hydrangeaceae	hydrangea	0.762	0.824
Hypericum	Hypericaceae	St. John’s wort	0.095	0.153
Impatiens	Balsaminaceae	jewelweed, touch-me-not	0.036	0.188
Melilotus*	Fabaceae	sweet-clover	0	0
Monarda	Lamiaceae	bee balm, wild bergamot	0	0
Nepeta	Lamiaceae	catmint	0	0
Pastinaca*	Apiaceae	wild parsnip	0	0.012
Plantago*	Plantaginaceae	plantain	0.06	0.118
Rhododendron	Ericaceae	rhododendron, azalea	0.19	0.259
Rosa	Rosaceae	wild rose	0.012	0.012
Rubus	Rosaceae	blackberry, raspberry	0.143	0.247
Rudbeckia*	Asteraceae	blackeyed Susan, coneflower	0.024	0.024
Securigera*	Fabaceae	crownvetch	0.048	0.235
Solidago*	Asteraceae	goldenrod	0.024	0.035
Symphyotrichum*	Asteraceae	aster	0.012	0.012
Taraxacum*	Asteraceae	dandelion	0.012	0
Thalictrum*	Ranunculaceae	meadow-rue	0.048	0.082
Trifolium*	Fabaceae	clover	0.083	0.141
Verbena	Verbenaceae	vervain	0	0.012
Vernonia	Asteraceae	ironweed	0.012	0.012

**Notes.**

† The ITS1 region was not represented in the database for this taxon.

## Discussion

Genetic analysis of individual corbicular pollen loads identified a diverse set of forage plants visited by *B. affinis* workers during late spring and summer in Appalachian sites of Virginia and West Virginia. These results document forage patterns otherwise difficult to observe directly, particularly for a rare species occupying terrain less suited to visual surveys than suburban or prairie habitats. The data should add confidence and nuance to science syntheses of *B. affinis* habitat requirements and the potential effects of management actions. While the plant genera identified in Appalachian pollen samples are often widespread within the historical range of *B. affinis*, *Rhododendron* and *Hydrangea* are far more common in Appalachia than the upper Midwest as noted above. These two genera plus *Actaea*, *Laportea* and *Tilia* were dominant in corbicular pollen samples from early to mid- summer but were largely absent in August. More qualitatively, a substantially lower proportion of genera identified in Appalachian samples overlapped with the large survey of Wolf et al. (2022) from the upper Midwest compared with the Midwestern samples analyzed identically (42.0% *vs.* 71.4%). Collectively, these observations confirm important regional differences in floral resources used by extant *B. affinis* populations.

Despite these regional differences, we were able to identify a consensus set of contemporary forage plants that transcended methodology and region. While such plants do not necessarily have an outsized role in supporting any given *B. affinis* population, it may be possible to formulate a broadly applicable index of habitat suitability based on their relative abundances at an appropriate scale. However, pollen data are predominantly available for workers; applying the methods used here to early spring foraging behavior may not be feasible despite its importance (Mola et al., 2021; Becher et al., 2024). This limitation is due to the greater rarity of such observations (see Fig. 1 of Wolf et al., 2022) as well as the potential detrimental effects of pollen sampling on nascent colonies.

A few unexpected plant genera were detected in this study. In particular, nettles of the genera *Laportea* and *Urtica* were detected at both loci (Fig. 5, File S2), *Laportea* abundantly so in Appalachian samples. These genera are primarily wind-pollinated, with inconspicuous flowers capable of explosive pollen release (Taylor, 2009). Small-bodied insects such as thrips have been documented on *Urtica* flowers (Taylor, 2009; James et al., 2015), but bumblebees have not been reported visiting them to our knowledge, despite opportunity to do so (*e.g.*, Dramstad & Fry, 1995). Validation of direct use by *B. affinis* is needed, as it is plausible that detections of these genera resulted from depositions of wind-borne pollen onto nearby plants. Yet nettles produce substantially less pollen than grasses and trees (Taylor, 2009) and would not be expected to greatly surpass those taxa as environmental contaminants. Furthermore, both *Laportea* and *Urtica* were detected by Simanonok et al. (2024) from corbicular pollen loads collected at the nest rather than in the field and processing of those samples occurred at a different facility, factors which disfavor laboratory contamination as an explanation. Another taxon detected unexpectedly frequently and at both loci was the mycoheterotrophic genus *Monotropa*. The genus was detected each year in a total of nine Appalachian samples (10.6%, Fig. 5). While known to be predominantly visited by bumblebees (Klooster & Culley, 2009), these nonphotosynthetic species require very specific associations with mycorrhizal fungi (Leake et al., 2004) and are generally rare in the landscape. *Monotropa* flowers may therefore be relatively attractive or rewarding to *B. affinis* workers where they occur. Direct observations of *B. affinis* visiting these taxa and validation of metabarcode sequences with palynological microscopy should be sought to confirm these interactions.

The higher plant diversity consistently seen in late-season Appalachian pollen raises several important questions regarding the management application of forage data. For example, it is not currently known whether high plant diversity in the diet is beneficial to *B. affinis*, for example by increasing the diversity of nutrients or reducing dependence on individual plant species. In fact, higher forage diversity could potentially indicate increased stress rather than increased nutrition, due to factors such as decreased overall resource availability, increased search effort, and decreased foraging efficiency (for example Heinrich, 1979). Furthermore, merely supplementing late season forage plants may not have the intended consequences for *B. affinis* populations if competing species extract a greater benefit. Pollen metabarcoding could potentially be used to study how temporal shifts in forage diversity of a focal insect species are shaped by changing plant-insect interactions at the community level (Kämper et al., 2016; Kolter et al., 2023; Hass et al., 2019), as well as their dependence on floral morphology and spatial structure (cf. Pornon et al., 2016). However, forage diversity of individual corbicular pollen loads may not predict forage diversity at the colony level and is uninformative of colony condition. Linking pollen forage behavior through the season to colony fate would therefore be logistically challenging and invasive for *B. affinis*, but might be tractable for an abundant proxy species.

This study demonstrates that host-plant “traplines”, *i.e.,* efficient survey routes with accessible populations of plants known to be visited by *B. affinis*, reveal far more plant interactions than the host species themselves (approximately five additional genera per sample on average with ITS2 primers). This diversity gain could not be assumed *a priori*, since foraging flights are typically a half hour to over two hours based on data for *Bombus terrestris* (Spaethe & Weidenmüller, 2002) and thus individuals collected early in a foraging bout should yield less pollen diversity on average than the cumulative diversity detectable upon return to the nest. Trapline surveys are logistically more feasible than finding and catching individuals near nests or in less accessible terrain, greatly increasing the number of useful samples obtained per unit survey cost. Conversely, the approach strongly weights prior experience and could systematically exclude the discovery of other floral resources used by *B. affinis*. Nonetheless, this study identified a high number of genera (most at both loci), a high percentage of the ‘consensus’ forbs listed in Table 1, and several rare or unexpected plant genera, all of which speak to the utility of this approach for descriptive as well as experimental work (*e.g.*, estimating land-use effects or evaluating land-management actions).

Although the *B. affinis* data presented here and elsewhere are consistent with generalism and high forage diversity at the population level (Wolf et al., 2022), as is characteristic of *Bombus* overall (but note Wood et al., 2019 regarding quantitative differences among species), the level of diversity inferred for individual pollen loads with metabarcoding may appear at odds with observational studies of individual worker behavior. Bumblebee workers are believed to show high levels of fidelity to a floral type once a reward is learned (Heinrich, 1976), at least until that resource is depleted (Heinrich, 1979). On the other hand, genetic detections are broadly consistent with microscopy (Smart et al., 2017; Richardson et al., 2021; Simanonok et al., 2023) and detection of multiple plant taxa per corbicular pollen sample has been reported by other investigators using microscopy and diverse genetic metabarcodes (*e.g.*, Gous et al., 2019; Arstingstall et al., 2021; Kolter & Gemeinholzer, 2021; Minahan & Brunet, 2025). It is possible that some taxonomic signal is due to wind- or animal-mediated transfer between heterospecifics plants rather than direct visitation by *B. affinis*. Primarily or exclusively wind-pollinated plants are indeed present in the current data (*e.g.*, *Pinus* as well as the nettle genera noted above) but at rates too low to explain this apparent diversity dilemma. While insect-mediated spread of heterospecific pollen is known to occur (Fang & Huang, 2013), it seems unlikely that the magnitude of heterospecific pollen encountered would be comparable in scale to the pollen legitimately produced by the host plants visited (excepting potentially on the stigmas themselves Fang & Huang, 2016). Even if individual pollen loads contain plant taxa not actively foraged by that individual bee, they remain a component of the diet and thus potentially relevant to management, particularly if those taxa are correlated with pathogen or pesticide exposure. Another consideration is that richness comparisons are potentially misleading when data sets differ greatly in scale, particularly in light of the challenge of separating rare legitimate taxa from low-level contamination in metabarcode data (*e.g.*, Edgar, 2018; Leray & Knowlton, 2017). Abundance-weighted diversity metrics such as Simpson’s or Shannon’s indexes may well be more consistent with visual observations. Regardless, quantifying the level of heterospecific pollen potentially encountered on plant taxa known to be visited by *B. affinis* is an important avenue of further research to aid the interpretation of metagenetic forage data. It is possible, for example, that the diversity of Asteraceae in late-season samples is exaggerated by frequent movement of insects between showy inflorescences of composite flowers with highly accessible pollen, in contrast to zygomorphic pea flowers, for example, a hypothesis that remains to be tested in these environments.

Despite these caveats, ITS2 metabarcoding continues to demonstrate good taxonomic resolution and high total diversity (Keller et al., 2015; Cornman et al., 2015; Gous et al., 2019; Macgregor et al., 2019; Richardson et al., 2021). Consistent with Gous et al., 2019, ITS1 performed less well, but not poorly, despite being frequently less represented in reference databases due to the historical focus on ITS2 (as was seen for *Ageratina* in this study). Both loci detected unique taxa, although most of these differences are likely due to stochastic detection of rare pollen sources rather than actual molecular biases between loci. In fact, two-thirds of genera detected in Appalachian samples were detected at both loci and dual detections were highly correlated with prevalence and overall sequence counts (Fig. 4). Nonetheless, primer bias likely explains the large differences in detection rates between loci for *Tilia* and *Daucus* and should be further examined with mock community analysis (discussed further below). On balance, we conclude that an optimized ITS2 protocol could be sufficient for many applications of pollen metabarcoding to the study of *B. affinis* ecology and conservation, which would reduce per-sample costs, but occasional checks with ITS1 or another locus such as *rbcL* (Espinosa Prieto et al., 2024) remain advisable, potentially on pooled samples for efficiency. However, ITS1 performed better than ITS2 in other *Bombus* metabarcoding work with respect to species-level resolution and total detections (Kolter et al., 2023), indicating that ITS marker choice remains an empirical determination that is context-dependent.

While we believe the minimum thresholds used for taxon detection in a sample are valuable for limiting false positives due to cross-talk and ‘natural’ contamination and are comparable to thresholds commonly used in the literature (Alberdi et al., 2018; Drake et al., 2022), we investigated how alternative thresholds could impact the results (File S4). Prevalence and Shannon’s H were re-evaluated while varying the minimum count threshold by an order of magnitude (1, 5, or 10) and the minimum relative abundance threshold by two orders of magnitude (0.01%, 0.1%, or 1%). Varying the minimum count threshold had negligible impact on prevalence. Varying the minimum relative abundance thresholds often had large effects on taxon prevalence, as expected when most taxa occur at low levels in samples and taxon proportions within a sample are inherently autocorrelated due to the compositional nature of metabarcoding data. Despite this, Shannon’s H remained highly consistent across this broad range of thresholds; values were appreciably reduced only at the highest relative abundance threshold of 1%, and predominantly in samples already at the low end of diversity.

A limitation of this study is that it did not include positive-control ‘mock’ communities of known composition. Diverse mock communities provide a basis for evaluating the performance of alternative primer sets for a locus *in vitro* for the community of interest (*e.g.*, Kolter & Gemeinholzer, 2021; Espinosa Prieto et al., 2024), which could deviate from *in silico* predictions. Mocks can also reveal biases in the recovery of individual taxa as well as in sample-level metrics such as diversity indices, and can be used to further tune data censoring approaches for cross-talk. Furthermore, mocks allow PCR conditions to be systematically evaluated, as many factors can affect relative and absolute recovery of taxa, which may not be readily predictable for complex mixtures. These factors include natural PCR inhibitors, differing characteristics of commercial polymerases and their reaction buffers, and an unlimited range of thermocycling profiles. Of particular note, plant taxa collectively have a broad range of GC contents (the proportion of bases that are guanine or cytosine) at ITS loci and templates with high GC content can be recalcitrant to PCR (Kolter & Gemeinholzer, 2021; Varadharajan & Parani, 2021). Amplification of high GC content templates can be improved with several potential additives to standard reaction buffers, including dimethyl sulfoxide (DMSO), glycerol, betaine, and proprietary additives, as well as by modulating magnesium chloride concentration and using repeated denaturing pulses during the extension phase of each PCR cycle (Green & Sambrook, 2019; Orpana, Ho & Stenman, 2012; Strien, Sanft & Mall, 2013; Guido et al., 2016; Tbahriti et al., 2025). Two of the three PCR steps used in library preparation were performed with ‘second generation’ polymerases that are rated for GC contents up to 70% according to the manufacturer’s specifications, however the initial preamplification reaction did not and likely resulted in dropout of high-GC plant taxa (see Kolter & Gemeinholzer, 2021). This limitation is suggested by a post hoc comparison of taxa identified in Appalachian samples in this study with taxa identified in the observational study of Hepner et al. (2024), which shows that high-GC taxa were disproportionately undetected by our metabarcoding protocol (Fig. S5). On the other hand, these taxa were also undetected at ITS1, which has substantially lower GC content than ITS2 (Fig. S5).

Custom reference database construction is an important component of metabarcoding workflows. We based our reference database primarily on the PLANiTS database, which is extensive, preformatted for SINTAX, and curated to exclude erroneous sequences (Banchi et al., 2020). The PLANiTS database clusters sequences at 99% within species for reduced computational effort but this approach has been shown to be less accurate for species-level assignments than an unclustered database (Kolter & Hebert, 2025). However, our analyses focused on the genus level such that any effect of reference clustering was likely reduced. We filtered our database at the genus level with a ten-state filter in the USDA Plants database. This database currently contains almost 100,000 taxon distributions and is credibly comprehensive for this region; it includes crop and invasive species as well as native species (https://plants.usda.gov/). While commercially distributed horticultural varieties are unlikely to be accurately documented in any database, the Appalachian samples were collected in National Forests and similar areas removed from agricultural or suburban landscapes. Thus, despite frequent roadside sample collection, false assignments caused by exotic horticultural varieties are unlikely to have impacted our results but should be considered in more developed areas where *B. affinis* colonies have been found (*e.g.*, Simanonok et al., 2024). Finally, it should be noted that the custom database used for taxonomic analysis was curated to represent the east-central portions of the historical range of *B. affinis*. Some plants restricted to the margins of this range may not have been represented. It would be worthwhile to further investigate with simulations the extent to which databases should be geographically constrained, recognizing that overly broad constraints can reduce taxonomic resolution whereas overly narrow constraints favor misassignment or unassignment. Inferring an optimal geographic scope of reference databases for *B. affinis* surveys could be complicated by regional and methodological variation in documenting plant distributions and regional biases in genetic sampling.

Given that numerous commonly visited taxa are closely related and the obvious appeal of species-level plant identifications to management, mock community analysis and simulated sequence data could help designate operational bins of species that are prone to conflation at a given locus based on the barcode gap (cf. Cornman, McKenna Jr & Fike, 2021). Binning is common in palynological microscopy (see studies cited herein) but infrequent in metabarcoding; rational delineation of taxon bins by metabarcode resolution rather than wholesale deferment to genus-level analyses could reduce error, improve comparability among studies, and allow more confident use of species-level inferences.

## Conclusions

In conclusion, two-locus metabarcoding of corbicular pollen samples supported a distinctive Appalachian forage for *B. affinis*, with the dominant taxa detected more prevalent in Appalachia than in the upper Midwest. Forage patterns indicate a generalist foraging strategy and an increase in forage diversity in late summer. A set of plant genera consistently detectable across methods and regions was identified and might be a useful metric of habitat suitability. While these data inform habitat management, they are inherently proportional and do not speak to the absolute abundance of forage plants on the landscape and how that relates to colony success.

##  Supplemental Information

10.7717/peerj.20284/supp-1 Supplemental Information 1 Reference sequence database used for taxonomic assignment of metagenetic sequences

10.7717/peerj.20284/supp-2 Supplemental Information 2 Raw and transformed sequence count data by assigned taxon and locus

10.7717/peerj.20284/supp-3 Supplemental Information 3 Comparison of genera identified as Bombus affinis forage plants in seven data sets Note that the studies used different methodologies and criteria for reporting identifications.

10.7717/peerj.20284/supp-4 Supplemental Information 4 Relation between minimum sequence abundance thresholds used to censor low-abundance data and two calculated variables, taxon prevalence and sample diversity (Shannon’s index, H) The thresholds evaluated are the absolute minimum count per taxon-sample combination (before aggregation at genus level) and the minimum proportion within a sample (applied after aggregation at the genus level). Three pairs of figures are shown; each pair contains data for ITS1 and ITS2. A figure legend is provided on each page. ITS1 = internal transcribed spacer 1, ITS2 = internal transcribed spacer 2.

10.7717/peerj.20284/supp-5 Supplemental Information 5 Taxa detected in the negative control sample are consistent with normal levels of demultiplex error rather than physical contamination The dashed line is the hypothesized maximum rate at which demultiplex error is expected to occur, points to the left of this line imply that physical contamination is likely. Demultiplex error at this level is addressed by censoring low-count cells and imposing a 0.1% threshold for detection at the genus level. ITS1 = internal transcribed spacer 1 and ITS2 = internal transcribed spacer 2.

10.7717/peerj.20284/supp-6 Supplemental Information 6 Output of the SINTAX taxonomic assignment algorithm for simulated sequence data, representing three scoring thresholds and three taxonomic ranks Read pairs were simulated from known references with realistic sequencing error profiles, trimmed, merged, and assigned with the same reference database (see text for details). When the simulated, merged read is given the same assignment as its source, it is considered a match. If the assignment does not match the source, it is considered an error. If no assignment is made at that rank and threshold score combination, the output is tabulated as “NA”. Any use of trade, firm, or product names is for descriptive purposes only and does not imply endorsement by the U.S. Government.

10.7717/peerj.20284/supp-7 Supplemental Information 7 Boxplots of Shannon’s diversity index H by calendar month of sampling (combined across years) Significant pairwise comparisons by Mann-Whitney test are show, Bonferroni-corrected for multiple tests within each locus. ITS1 = internal transcribed spacer 1 and ITS2 = internal transcribed spacer 2.

10.7717/peerj.20284/supp-8 Supplemental Information 8 Proportion of plant genera unique to a *Bombus affinis* forage survey data set relative to the total number of genera detected Appalachian and Midwestern samples from the current study are indicated, with short citations indicating previously published work. Capital letters following each data set indicate study methodology (M = metabarcoding, P = palynological microscopy, O = observation). The complete study citations are: Hepner MJ, Orcutt E, Price K, Goodell K, Roulston T, Jean RP, Richardson RT. 2024. Montane Central Appalachian forests provide refuge for the critically endangered rusty patched bumble bee (*Bombus affinis*). Forest Ecology and Management 556:121751. DOI: 10.1016/j.foreco.2024.121751; Simanonok MP, Evans E, Otto CR, Cornman RS, Iwanowicz DD, Smith TA. 2024. Floral Composition of Pollen Collected from a Rusty Patched Bumble Bee (*Bombus affinis*, Cresson) Nest in Southeastern Minnesota. Prairie Naturalist 56:27–41. Available at: https://pubs.usgs.gov/publication/70257059; Simanonok MP, Iwanowicz DD, Raines CD, Wood TJ, Isaacs R, Cornman RS, Otto CR. 2023. Comparison of microscopy and metabarcoding to identify pollen used by the critically endangered rusty patched bumble bee, Bombus *affinis*. Insect Conservation and Diversity 16:205–216. DOI: 10.1111/icad.12622; Simanonok MP, Otto CR, Cornman RS, Iwanowicz DD, Strange JP, Smith TA. 2021. A century of pollen foraging by the endangered rusty patched bumble bee (*Bombus affinis*): inferences from molecular sequencing of museum specimens. Biodiversity and Conservation 30:123–137. DOI: 10.1007/s10531-020-02081-8; Wolf AT, Watson JC, Hyde TJ, Carpenter SG, Jean RP. 2022. Floral resources used by the endangered rusty patched bumble bee (*Bombus affinis*) in the Midwestern United States. Natural Areas Journal 42:301–312. DOI: 10.3375/22-2

10.7717/peerj.20284/supp-9 Supplemental Information 9 Average GC content of full-length amplicons of each metabarcode locus extracted from the reference database, for plant genera found in a published visual survey of *Bombus affinis* forage plants in the Appalachian region Taxa are grouped according to whether they were detected in this study in Appalachian samples (see text for details). GC = guanine plus cytosine; ITS1 = internal transcribed spacer 1; ITS2 = internal transcribed spacer 2.

## Additional Information and Declarations

### Field Study Permissions

The following information was supplied relating to field study approvals (*i.e.*, approving body and any reference numbers): Collections were made under the following permits where required by state and federal agencies: ES61005D (U.S. Fish and Wildlife Service); 2021.085, 2022.113, 2023.196 (West Virginia Department of Natural Resources); 4200 (Monongahela National Forest); 2600, 2670 (George Washington and Jefferson National Forests); ES02373A-16 (U.S. Fish and Wildlife Service).

### DNA Deposition

The following information was supplied regarding the deposition of DNA sequences: Sequence data generated by this study have been deposited in the Sequence Read Archive of the National Center for Biotechnology Information under accession PRJNA1235776. Accessions and metadata are also provided as a USGS data release, https://doi.org/10.5066/P1FOTKNB.

### Data Availability

The following information was supplied regarding data availability: The sequences are available at NCBI: PRJNA1235776.
