# Supplementary material for: The Appalbees menu: a multiyear, multilocus metagenetic assessment of pollen foraging by Appalachian Bombus affinis workers
Source: PeerJ. 2026 Jan 12;14:e20284. doi: 10.7717/peerj.20284 (PMC12805910; doi:10.7717/peerj.20284)

Supplemental File S4. Relation between minimum sequence abundance thresholds used to censor low-abundance data and two calculated variables, taxon prevalence and sample diversity (Shannon's index, H). The thresholds evaluated are the absolute minimum count per taxon-sample combination (before aggregation at genus level) and the minimum proportion within a sample (applied after aggregation at the genus level). Three pairs of figures are shown; each pair contains data for ITS1 and ITS2. A figure legend is provided on each page. ITS1 = internal transcribed spacer 1, ITS2 = internal transcribed spacer 2.

Legend: Prevalence of each taxon under different minimum thresholds of absolute sequence count in each cell of the taxon-sample matrix. The value used in the text is ten and is compared to alternative values of five and one (i.e., no minimum count). The relative abundance threshold used in the text, 0.001, is held constant in this comparison.

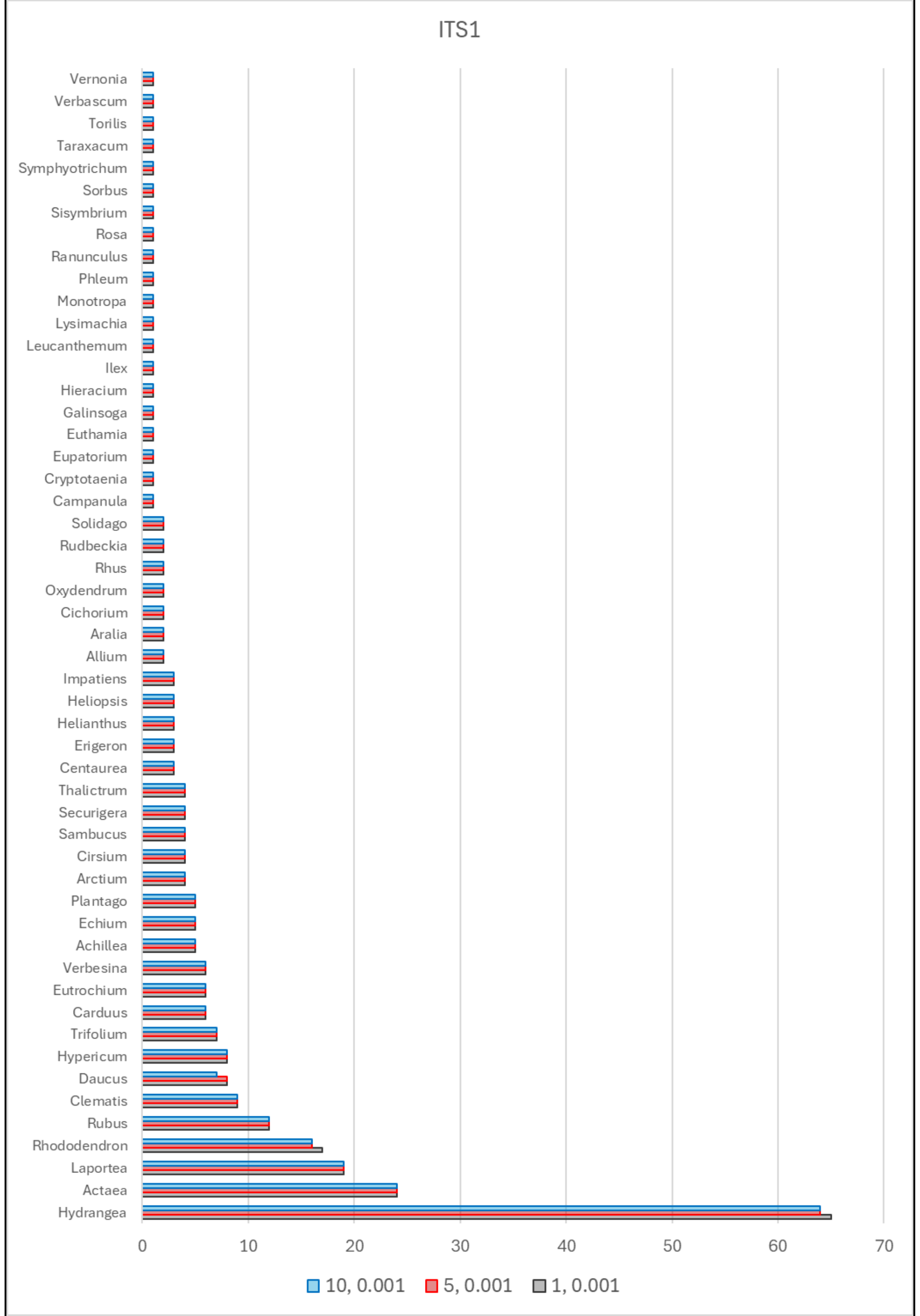

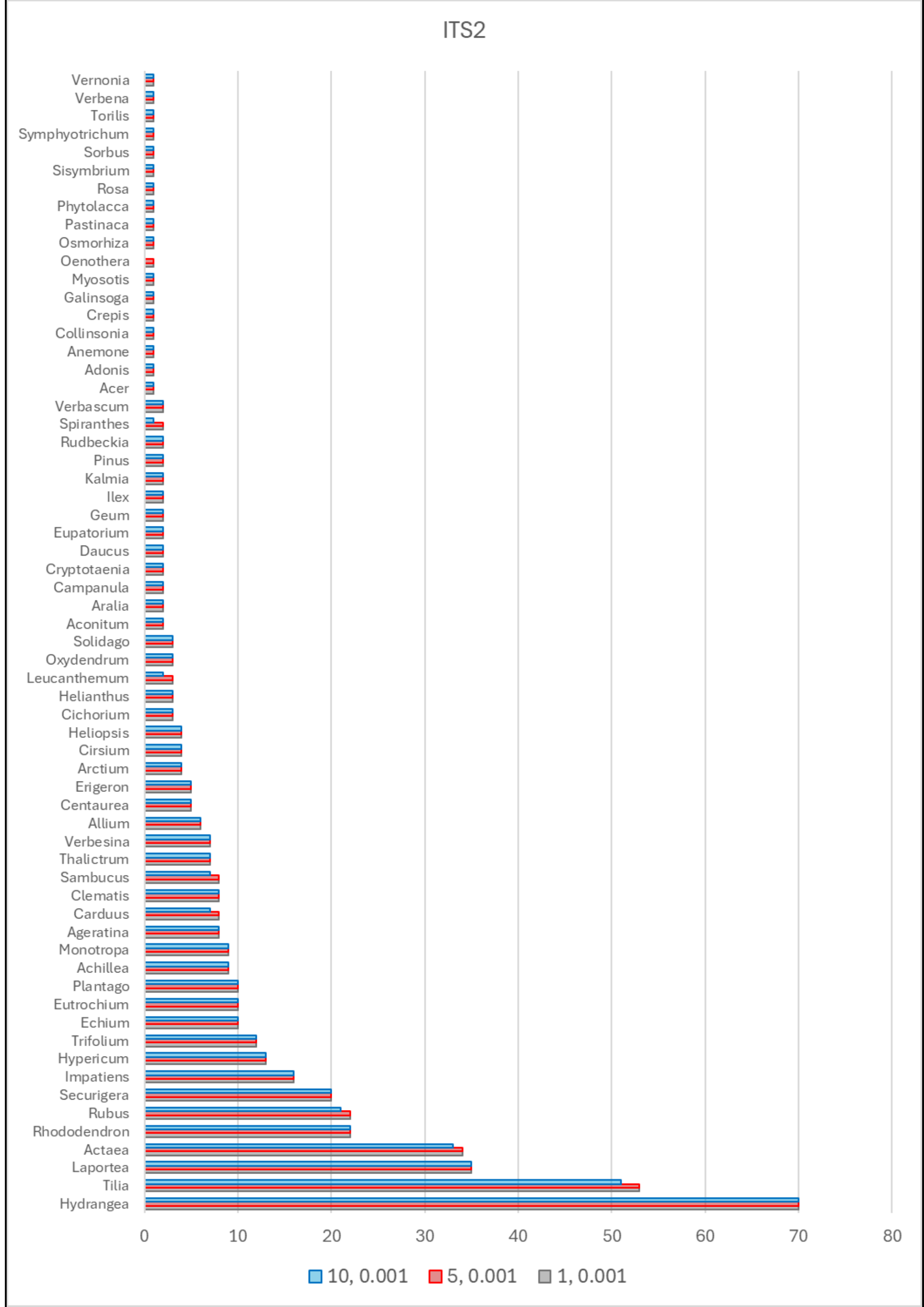

Legend: Prevalence of each taxon under different minimum thresholds of relative sequence abundance in each cell of the taxon-sample matrix. The value used in the text is 0.001 and is compared to alternative values of 0.0001 and 0.01. The absolute minimum abundance used in the text, 10, is held constant in this comparison.

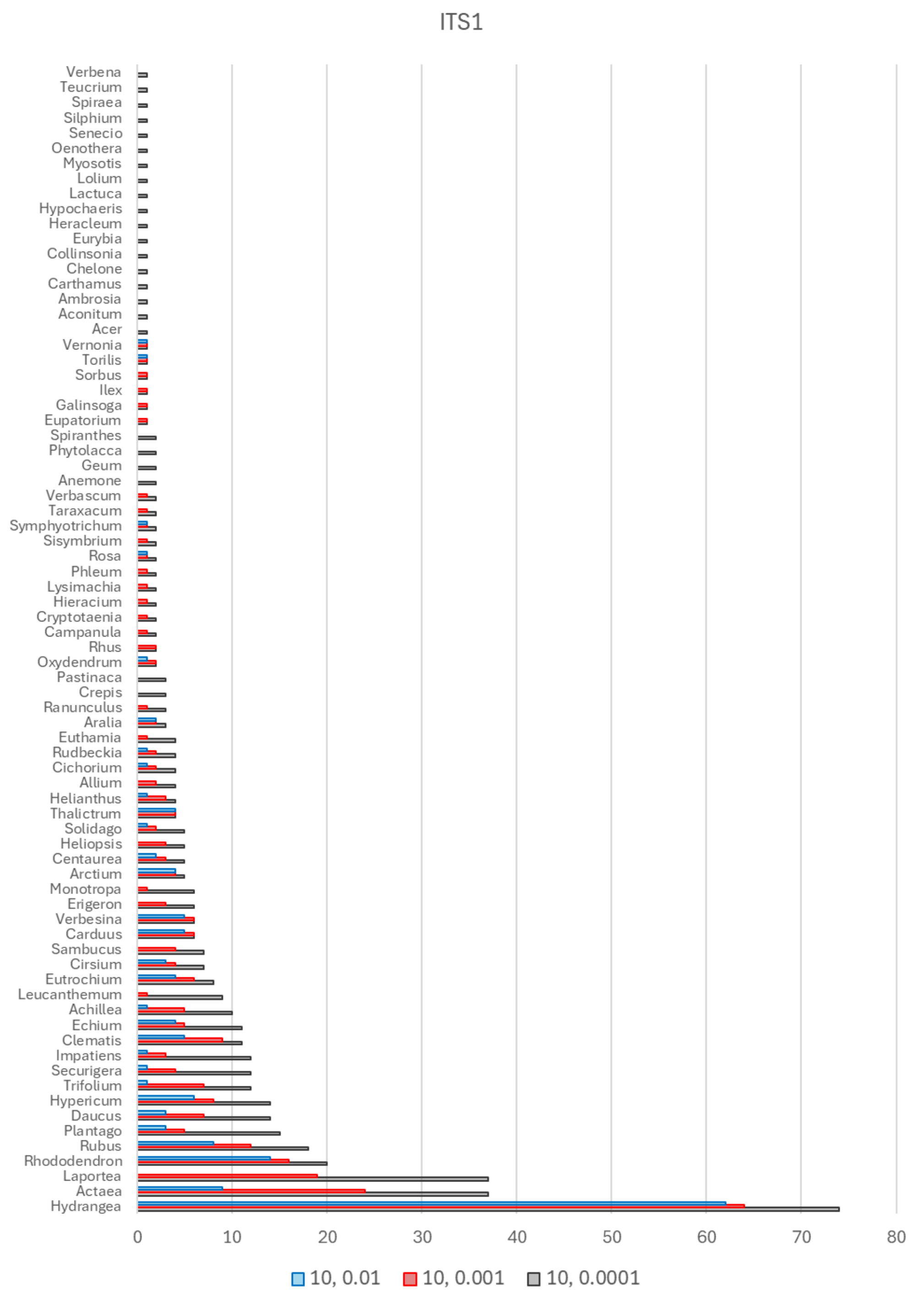

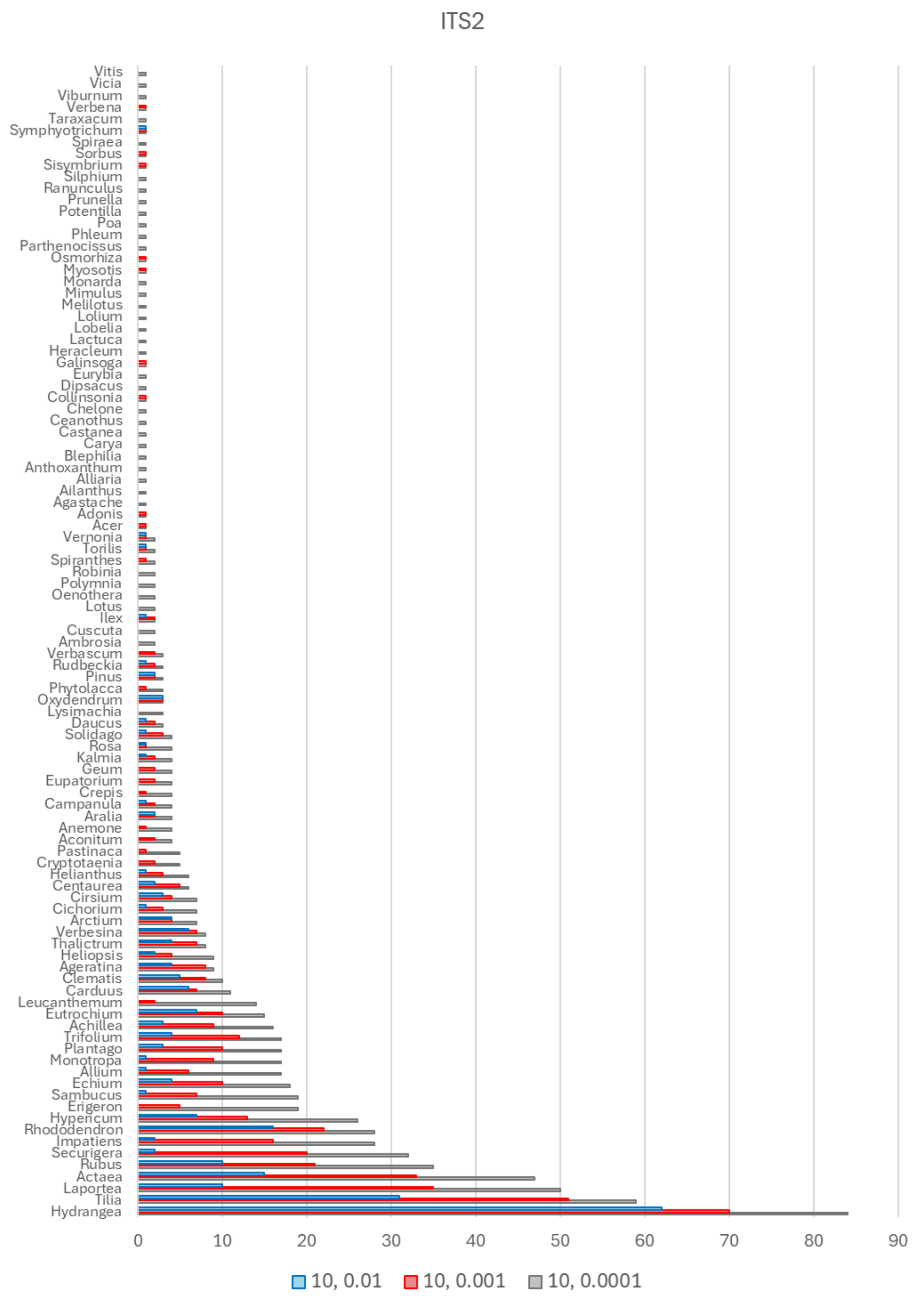

Legend: Shannon diversity index H of each sample under different minimum thresholds of relative sequence abundance in each cell of the taxon-sample matrix. The value used in the text is 0.001 and the H estimates for samples under this threshold are correlated with estimates under alternative values of 0.0001 and 0.01. No minimum abundance is applied as that would obscure the effect of varying the relative threshold.

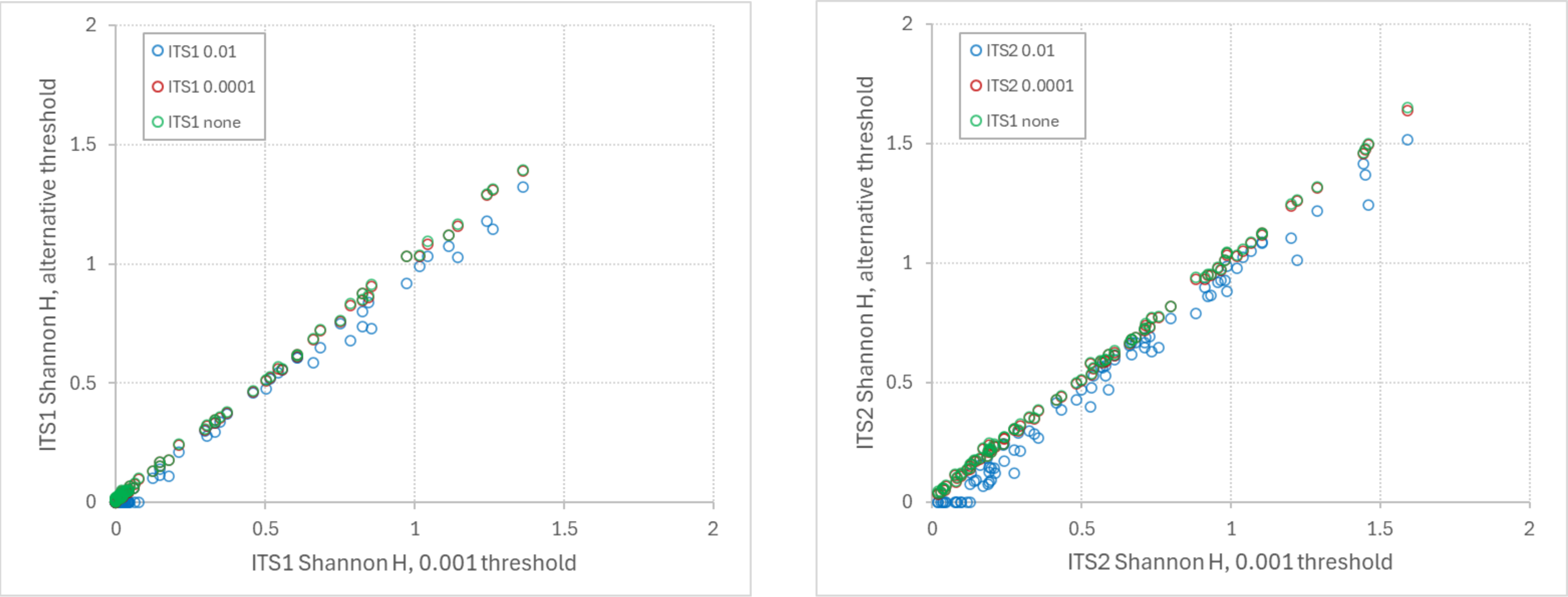

Supplement: Supplemental Information 4 — The thresholds evaluated are the absolute minimum count per taxon-sample combination (before aggregation at genus level) and the minimum proportion within a sample (applied after aggregation at the genus level). Three pairs of figures are shown; each pair contains data for ITS1 and ITS2. A figure legend is provided on each page. ITS1 = internal transcribed spacer 1, ITS2 = internal transcribed spacer 2. [file peerj-14-20284-s004.pdf]
