# Supplementary figures and images for: The Appalbees menu: a multiyear, multilocus metagenetic assessment of pollen foraging by Appalachian Bombus affinis workers
Source: PeerJ. 2026 Jan 12;14:e20284. doi: 10.7717/peerj.20284 (PMC12805910; doi:10.7717/peerj.20284)

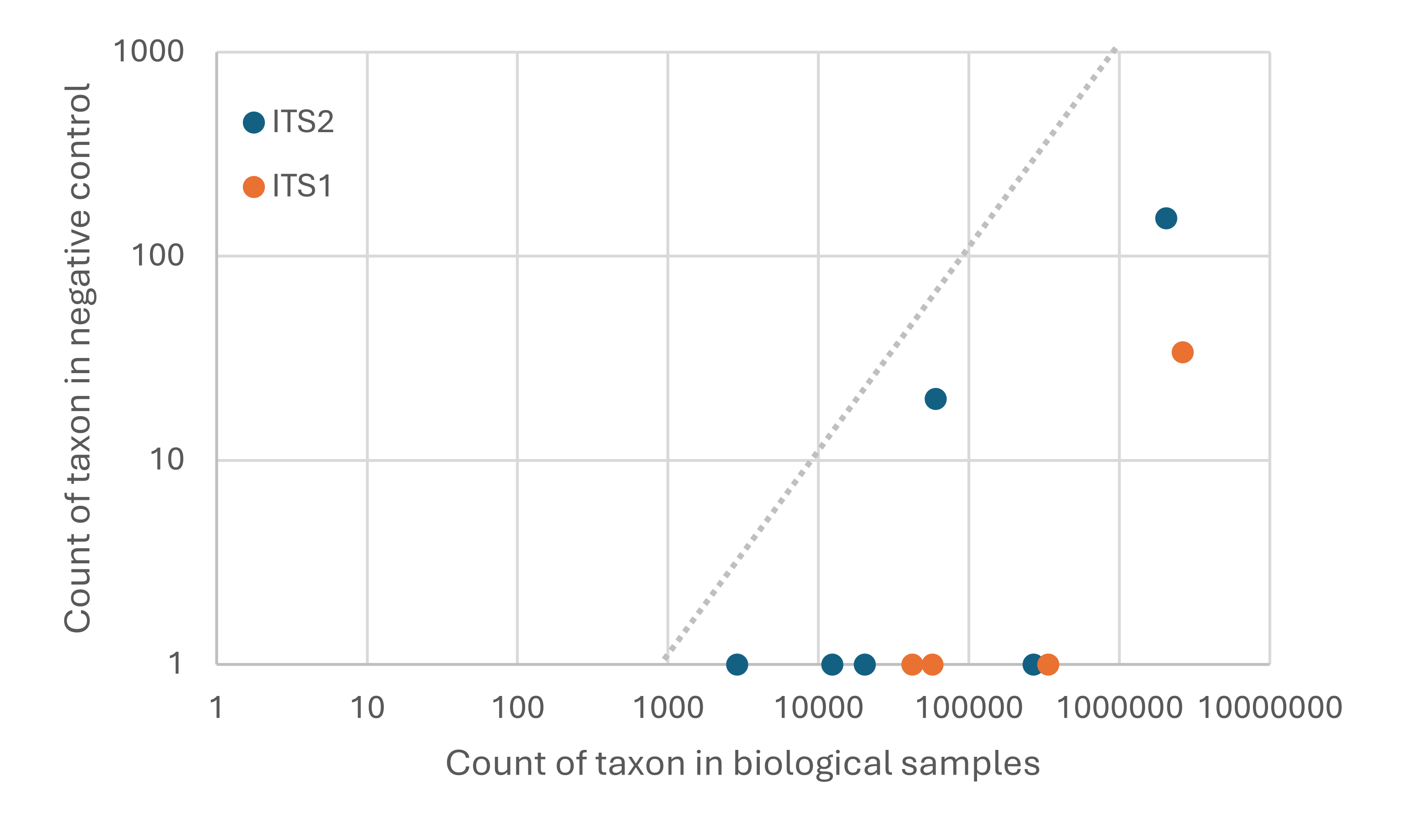

Supplement: Supplemental Information 5 — The dashed line is the hypothesized maximum rate at which demultiplex error is expected to occur, points to the left of this line imply that physical contamination is likely. Demultiplex error at this level is addressed by censoring low-count cells and imposing a 0.1% threshold for detection at the genus level. ITS1 = internal transcribed spacer 1 and ITS2 = internal transcribed spacer 2. [file peerj-14-20284-s005.png]

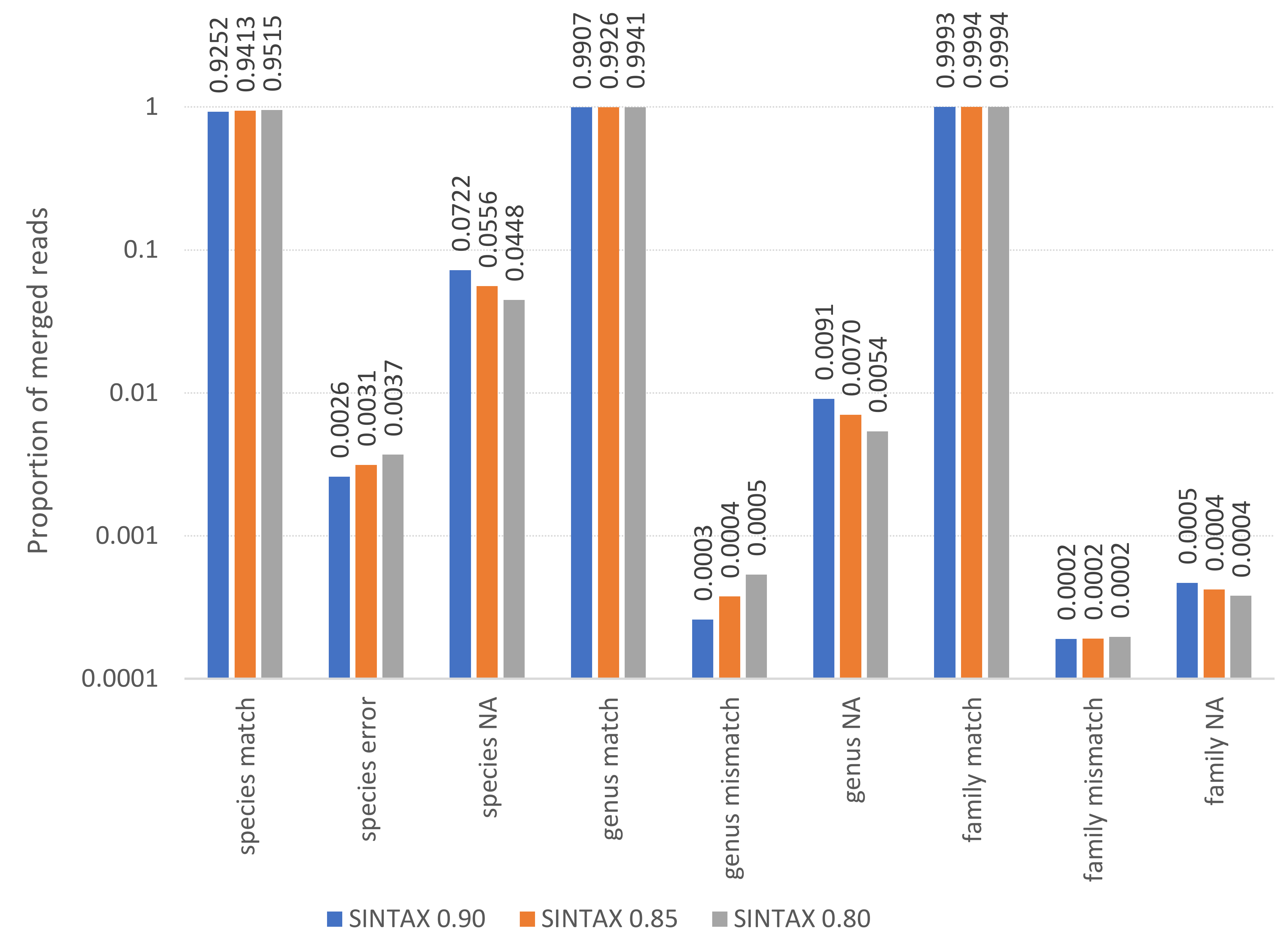

Supplement: Supplemental Information 6 — Read pairs were simulated from known references with realistic sequencing error profiles, trimmed, merged, and assigned with the same reference database (see text for details). When the simulated, merged read is given the same assignment as its source, it is considered a match. If the assignment does not match the source, it is considered an error. If no assignment is made at that rank and threshold score combination, the output is tabulated as “NA”. Any use of trade, firm, or product names is for descriptive purposes only and does not imply endorsement by the U.S. Government. [file peerj-14-20284-s006.png]

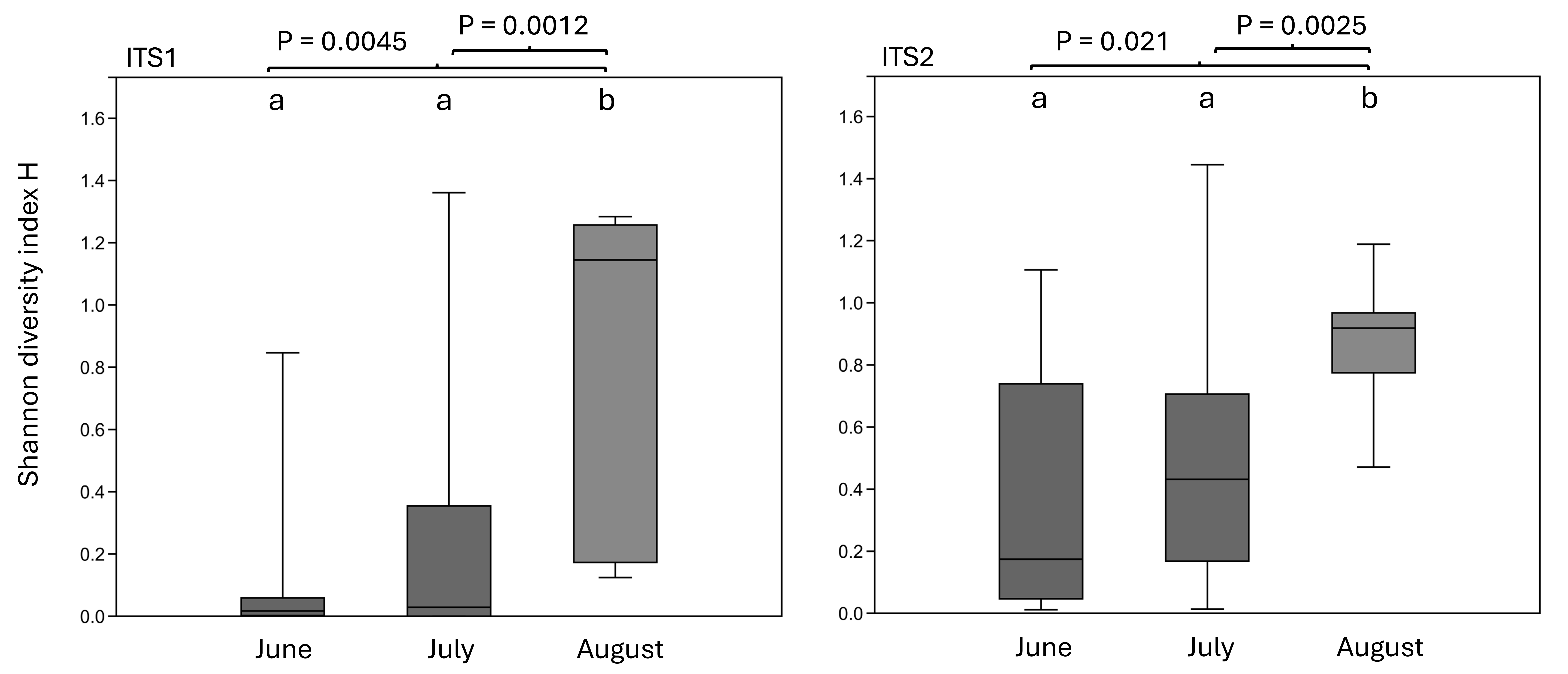

Supplement: Supplemental Information 7 — Significant pairwise comparisons by Mann-Whitney test are show, Bonferroni-corrected for multiple tests within each locus. ITS1 = internal transcribed spacer 1 and ITS2 = internal transcribed spacer 2. [file peerj-14-20284-s007.png]

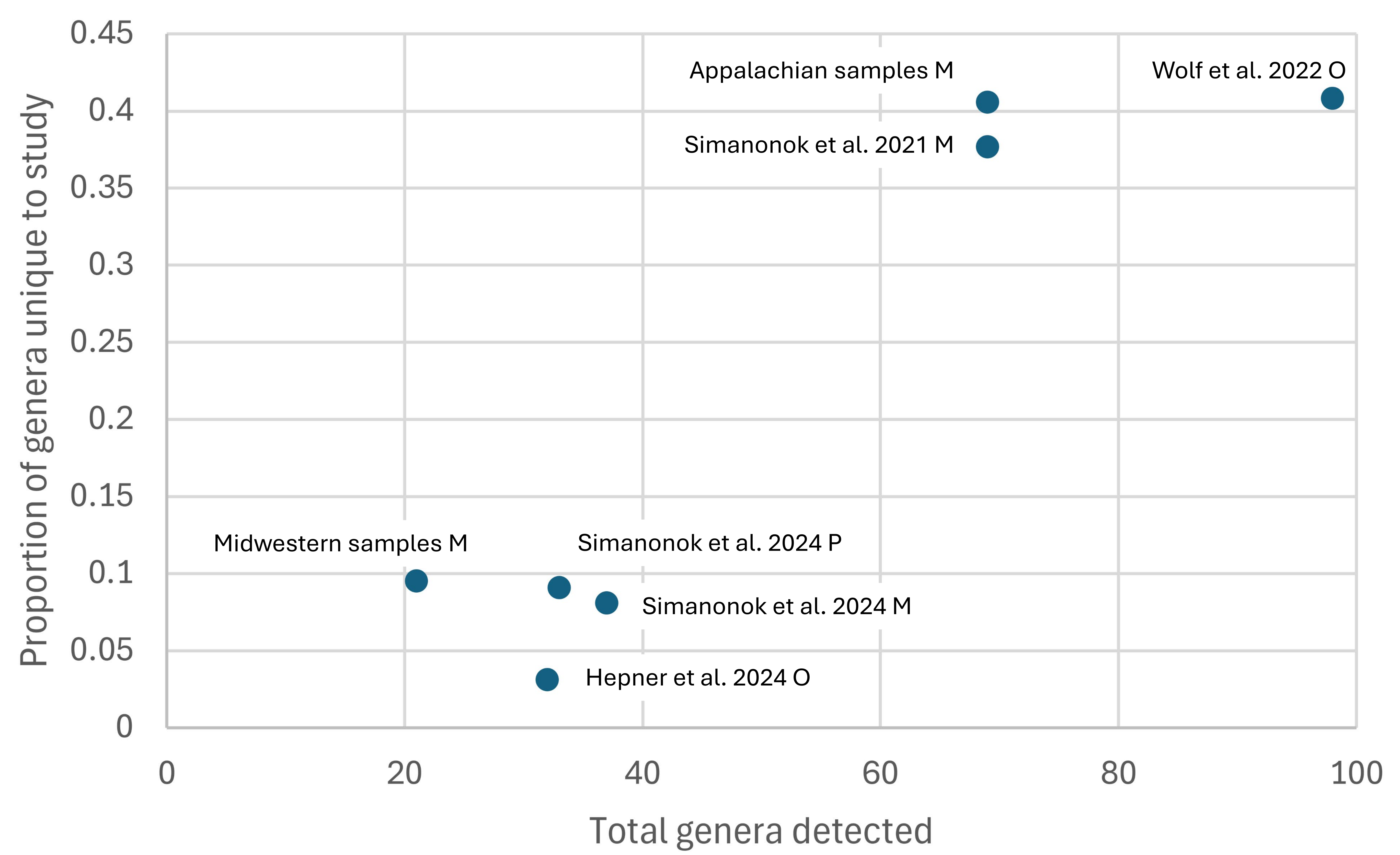

Supplement: Supplemental Information 8 — Appalachian and Midwestern samples from the current study are indicated, with short citations indicating previously published work. Capital letters following each data set indicate study methodology (M = metabarcoding, P = palynological microscopy, O = observation). The complete study citations are: Hepner MJ, Orcutt E, Price K, Goodell K, Roulston T, Jean RP, Richardson RT. 2024. Montane Central Appalachian forests provide refuge for the critically endangered rusty patched bumble bee (Bombus affinis). Forest Ecology and Management 556:121751. DOI: 10.1016/j.foreco.2024.121751; Simanonok MP, Evans E, Otto CR, Cornman RS, Iwanowicz DD, Smith TA. 2024. Floral Composition of Pollen Collected from a Rusty Patched Bumble Bee (Bombus affinis, Cresson) Nest in Southeastern Minnesota. Prairie Naturalist 56:27–41. Available at: https://pubs.usgs.gov/publication/70257059; Simanonok MP, Iwanowicz DD, Raines CD, Wood TJ, Isaacs R, Cornman RS, Otto CR. 2023. Comparison of microscopy and metabarcoding to identify pollen used by the critically endangered rusty patched bumble bee, Bombus affinis. Insect Conservation and Diversity 16:205–216. DOI: 10.1111/icad.12622; Simanonok MP, Otto CR, Cornman RS, Iwanowicz DD, Strange JP, Smith TA. 2021. A century of pollen foraging by the endangered rusty patched bumble bee (Bombus affinis): inferences from molecular sequencing of museum specimens. Biodiversity and Conservation 30:123–137. DOI: 10.1007/s10531-020-02081-8; Wolf AT, Watson JC, Hyde TJ, Carpenter SG, Jean RP. 2022. Floral resources used by the endangered rusty patched bumble bee (Bombus affinis) in the Midwestern United States. Natural Areas Journal 42:301–312. DOI: 10.3375/22-2 [file peerj-14-20284-s008.png]

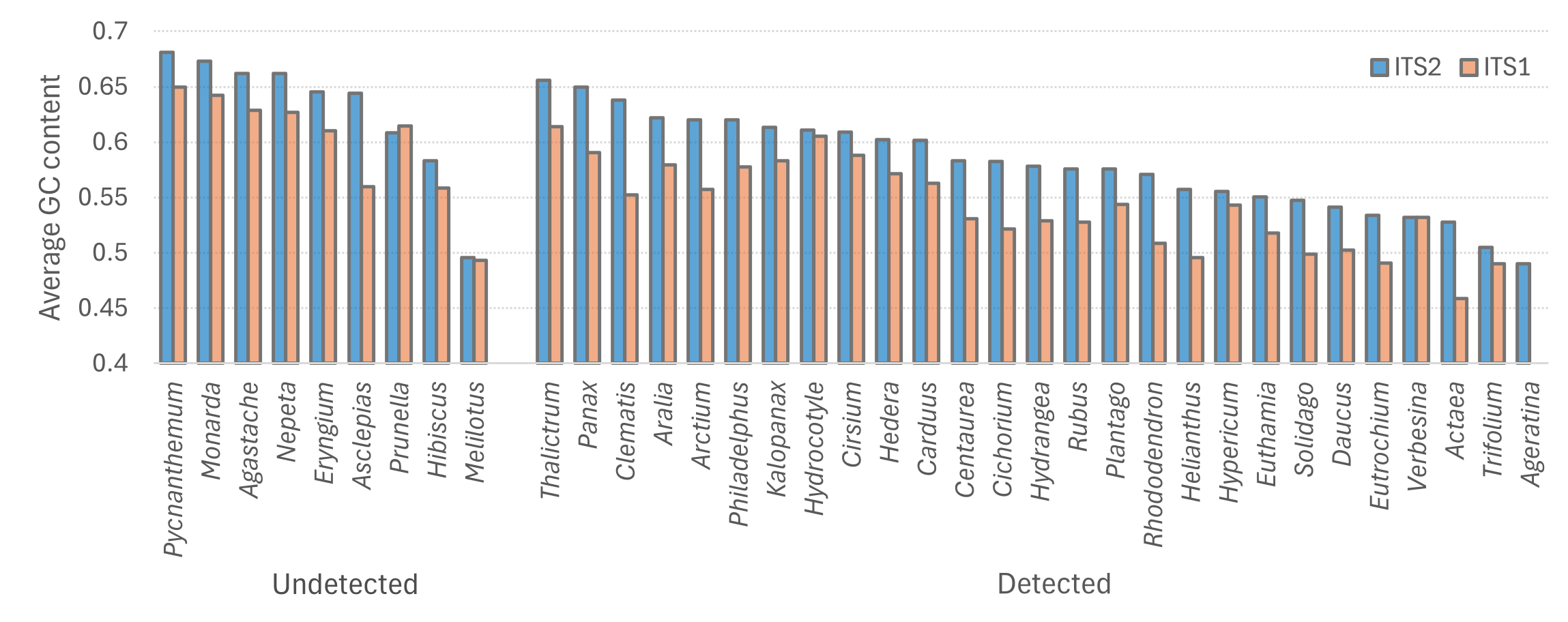

Supplement: Supplemental Information 9 — Taxa are grouped according to whether they were detected in this study in Appalachian samples (see text for details). GC = guanine plus cytosine; ITS1 = internal transcribed spacer 1; ITS2 = internal transcribed spacer 2. [file peerj-14-20284-s009.png]
